# Utilization of Algerian calcined clay in sustainable mortars considering thermal treatment and granulometry effects on mechanical properties

**DOI:** 10.1038/s41598-025-04342-4

**Published:** 2025-07-01

**Authors:** Mouna Badaoui, Kamel Hebbache, Abdellah Douadi, Lamia Mansouri, Abdelghani Merdas, Soria Gherbi, Karima Kada, Mourad Boutlikht, Cherif Belebchouche, Jacek Szymanowski

**Affiliations:** 1 Emerging Materials Research Unit (URME), Department of Civil Engineering, Ferhat Abbas University of Sétif 1, 19000 Sétif, Algeria; 2 Civil Engineering Research Laboratory of Sétif (LRGCS), Department of Civil Engineering, Setif 1 University-Ferhat Abbas, 19000 Sétif, Algeria; 3 Department of Process Engineering, Setif 1 University-Ferhat Abbas, 19000 Sétif, Algeria; 4 Materials and Durability of Constructions Laboratory, Faculty of Sciences of Technology, University of Constantine 1 Frères Mentouri, 25000 Constantine, Algeria; 5 Department of Materials Engineering and Construction Processes, Faculty of Civil Engineering, Wrocław University of Science and Technology, Wrocław, Poland

**Keywords:** Pozzolan, Raw clay, Calcined clay, Flexural strength, Compressive strength

## Abstract

This study investigates the potential of calcined Algerian clay as a supplementary cementitious material (SCM) for enhancing the sustainability of cement production by reducing the consumption of clinker and emissions of carbon dioxide (CO₂). The research novelty lies in the comprehensive evaluation of fineness effects, the statistical modelling of mechanical performance, and the assessment of the environmental impact. A case study of practical significance is also provided. Clay was thermally activated at 700 °C to achieve near-complete dihydroxylation, ground to fineness levels of 45 µm and 80 µm, and then incorporated into the cement at replacement ratios of 10–30% by weight of the cement. Compressive and flexural strengths were assessed at 2, 7, and 28 days, while thermal and structural modifications were analysed using thermogravimetric analysis (TGA) and Fourier-transform infrared spectroscopy (FTIR). The results demonstrated that at lower substitution levels (10–15%), the 80 µm fraction of clay enhanced early-age compressive strength (13.6 MPa at 2 days), whereas at higher replacements (20–30%), the 45 µm fraction exhibited superior long-term strength (36.85 MPa at 28 days), which was attributed to improved pozzolanic reactivity and matrix densification. Flexural strength increased by 7–20% for substitution rates up to 20%, in turn confirming the structural benefits of calcined clay. Life-cycle analysis indicated significant reductions in energy consumption, CO₂ emissions, and production costs, with the M30–45 and M30–80 mixtures achieving reductions of approximately 10.6%. The statistical modeling of compressive strength at 2 and 28 days demonstrated high predictive accuracy, with coefficients of determination (R^2^) of 0.93 and 0.81, respectively. These models were statistically validated using analysis of variance (ANOVA), confirming their significance at a 95% confidence level (*p* < 0.05). These findings showed calcined Algerian clay to be a viable SCM, with it demonstrating enhanced mechanical performance, environmental sustainability, and economic feasibility, thereby contributing to the decarbonization of the cement industry.

## Introduction

Cement production is highly energy-intensive and represents one of the major sources of global carbon dioxide (CO_2_) emissions, accounting for approximately 5% to 7% worldwide^1^. The use of mineral additions, especially pozzolanic materials, as a partial substitution for clinker in cement or Portland cement in concrete and mortar is a very promising solution^2–6^. According to ASTM C125, a pozzolan is defined as a siliceous or siliceous and aluminous material which, although possessing little or no cementitious value on its own, can chemically react with calcium hydroxide in the presence of moisture at ambient temperature, forming compounds with cementitious properties^7^. Artificial pozzolans are derived from a variety of sources, including natural materials such as finely ground sand^8^ and waste clays^9^. Additionally, byproducts such as silica fume and fly ash^10^, as well as industrial and agricultural wastes such as bagasse ash^11^, rice husk ash^12^ and fly ash^13^, are used for this purpose. However, these materials require mechanical (very fine grinding), chemical (alkaline attack), or hydrothermal treatment to exhibit pozzolanic activity^14^. Table 1 shows the different pozzolanic materials that are used as substitution materials.

**Table 1:** Different pozzolanic materials used as substitution materials of cement.

Activation method	Studied parameters	Main results
Grinding^15^	Replacement ratio of natural pozzolan (0 to 35%)	Increasing the amount of added natural pozzolan significantly reduces the early age strength, improves workability, and temporarily delays the setting time
Hydrothermal processing^16^		Thermogravimetry results revealed a significant increase in the reactivity of natural pozzolans as a result of the hydrothermal process
Thermal treatment and grinding	Temperature (600, 700, 800 °C), holding time (2, 3, 5 h) and replacement ratio of clay^9^	Heating for 5 h at 700 °C. The blended cement prepared with 10% calcined clay presents the highest strength and the lowest porosity and water absorption
	Type and amount of clayed minerals and the pozzolanic activity assessment methods^17^	A good correlation between the compressive strength and the content of argillaceous minerals in clays
Hydrothermal processing^14^	Replacement ratio of natural kaolin (0 to 20% w)	The hydrothermal technique is an effective method for the nanomodification of natural pozzolans
Grinding^8^	Replacement ratio (0, 10, 15, and 20%), and fineness (20, 40, and 80 µm) of sand	The optimum effect on compressive strengths was obtained when the replacement ratio was 10% and the fineness was 20 µm
Use of silica fume (SF) as it is^18^. Grinding coarse SF	Effect of silica fume (SF) fineness on pozzolanicity	SF with high fineness is suitable for making CEM II/A–D cement when percentages of SF are lower than 10%
No treatment	Fineness of fly ash (FA)^13^	The fineness of fly ash is one of the most important properties affecting pozzolanic activity. The highest compressive strengths were obtained when the replacement ratio was equal to 10%
No treatment	Substitution ratio (10, 20, and 30%) of fly ash (FA)^11^	Blending cement with 20% of FA is beneficial
Chemical and mechanical activation^19^	Substitution ratio of fly ash (FA)	Combining mechanical and chemical activation can be seen as an effective approach for enhancing early strength and slow strength development
No treatment	Substitution ratio of micro ceramic powder (MCP) (10, 20, 30, and 40%)^20^	A 20% replacement of cement with MCP resulted in an economic gain of 9.6% in concrete production and a 6.62% reduction in specific energy consumption during cement manufacturing
Calcination and grinding	Electrical conductivity and kinetic parameters of silica fume and bamboo leaf ash (BLAsh)^21^	Both silica fume and BLAsh have a similar reactivity. The use of this agricultural residue in the production of blended cements is of significant importance
Drying and grinding	Substitution ratio of rice husk ash (RHA)^22^	RHA is a suitable pozzolanic material for Portland pozzolan cement production
	Effect of rice husk ash (RHA) substitution ratio on the workability of concrete^12^	The incorporation of RHA in concrete increased water demand. The compressive strength of the blended concrete with 10% of RHA increased significantly
No treatment	Substitution ratio of bagasse ash (BA) (10, 20, 30%)^11^	The physical and chemical properties of BA meet the requirements as a pozzolanic material. Blending cement with 10% of BA is beneficial
Drying and grinding	Substitution ratio of date palm ash (DPA) (10, 20, 30%)^23^	A 10% replacement of OPC with DPA provides a strong and durable alternative, while increasing the substitution level up to 30% allows for greater clinker reduction with acceptable performance
Grinding	Fineness of glass powder^24^	Regardless of the colour of the samples, waste glass with a particle size lower than 40 µm is beneficial as a partial replacement of Portland cement

Based on Table 1, various types of natural pozzolans are gaining increasing attention due to their economic, environmental, and technical advantages. Their incorporation into cementitious materials contributes to a reduction in CO₂ emissions^7^, a lowering of the energy consumption during clinker production, and the promotion of efficient waste management^25^. Additionally, pozzolanic materials influence the physical and mechanical properties of cement-based systems^7,26,27^, notably by refining pore structure and decreasing permeability, which in turn enhances durability and long-term mechanical performance.

**Fig. 1:**
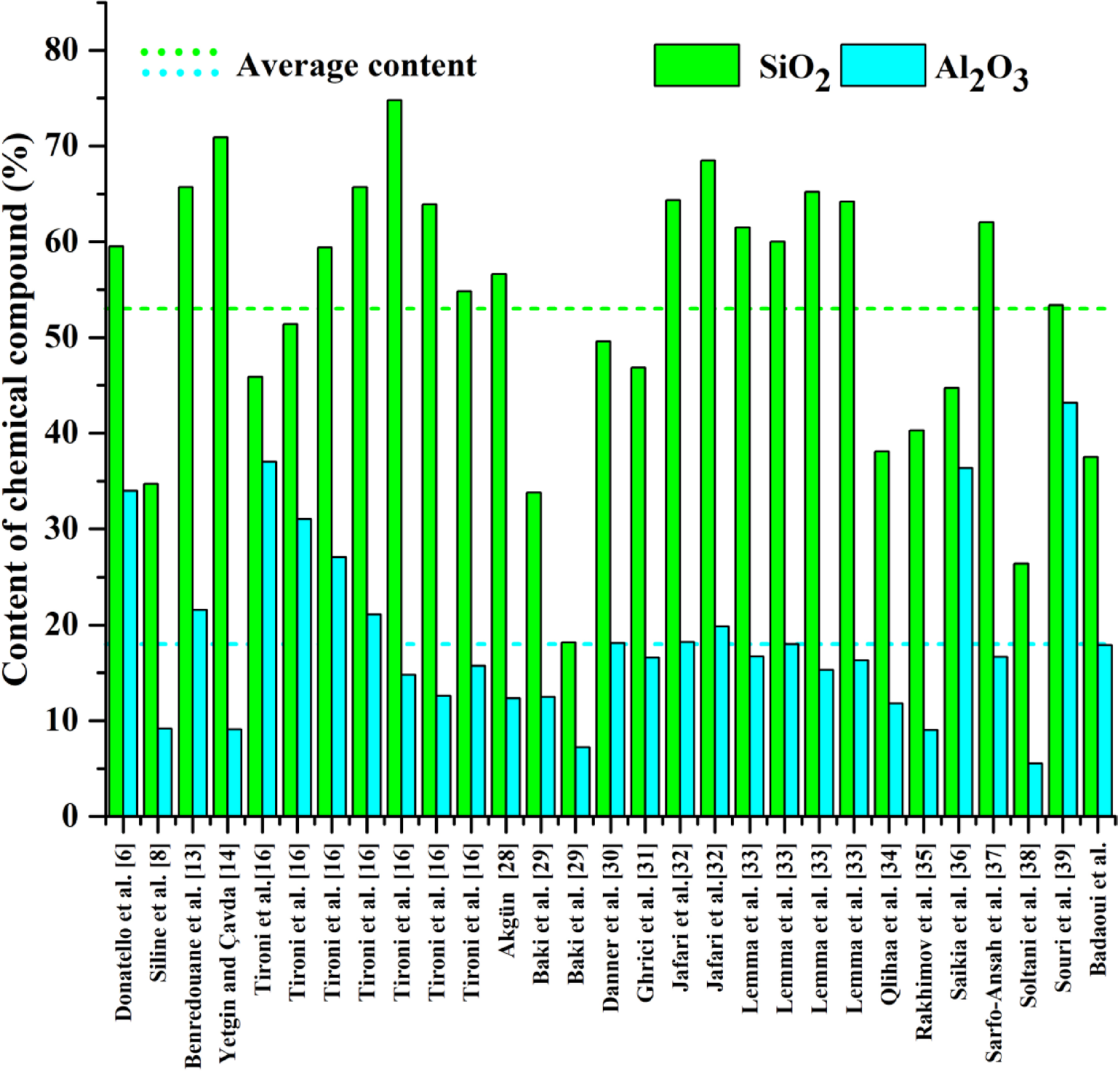
The silicon and aluminum oxide content in the raw clay presented in the literature^7,9,14,15,17,32–43^.

Among these materials, calcined clay has emerged as a scientifically substantiated alternative for mitigating the environmental impact of Portland cement. Its use as a supplementary cementitious material (SCM) enables a significant reduction in clinker content—the primary source of CO₂ emissions in cement manufacturing. Unlike clinkerization, which requires high temperatures (~1450°C), the thermal activation of kaolinitic clays at moderate temperatures (600–800°C) demands substantially less energy, leading to a notable decrease in energy consumption and a lower carbon footprint. Furthermore, the pozzolanic reactivity of calcined clay accelerates cement hydration, contributing to a denser microstructure that has enhanced durability, reduced permeability, and improved resistance to aggressive environments. These benefits align with key United Nations Sustainable Development Goals (SDGs). SDG 9 (Industry, Innovation, and Infrastructure) is addressed by fostering advancements in sustainable construction materials and promoting eco-efficient industrial processes^28^. SDG 11 (Sustainable Cities and Communities) is supported through the development of more durable and environmentally friendly concrete, in turn enhancing the resilience of urban infrastructure^29^. SDG 12 (Responsible Consumption and Production) is reinforced by optimizing resource efficiency, minimizing the reliance on energy-intensive clinker production, and utilizing locally available materials, thereby reducing the depletion of natural resources^30^. Finally, SDG 13 (Climate Action) is directly targeted by mitigating global warming through CO₂ emission reductions in cement production^31^. In this context, Figure 1 presents the silicon dioxide (SiO₂) and aluminum oxide (Al₂O₃) content in the raw clay used in various studies, and highlights their role in determining pozzolanic activity and hydration behaviour.

**Fig. 2:**
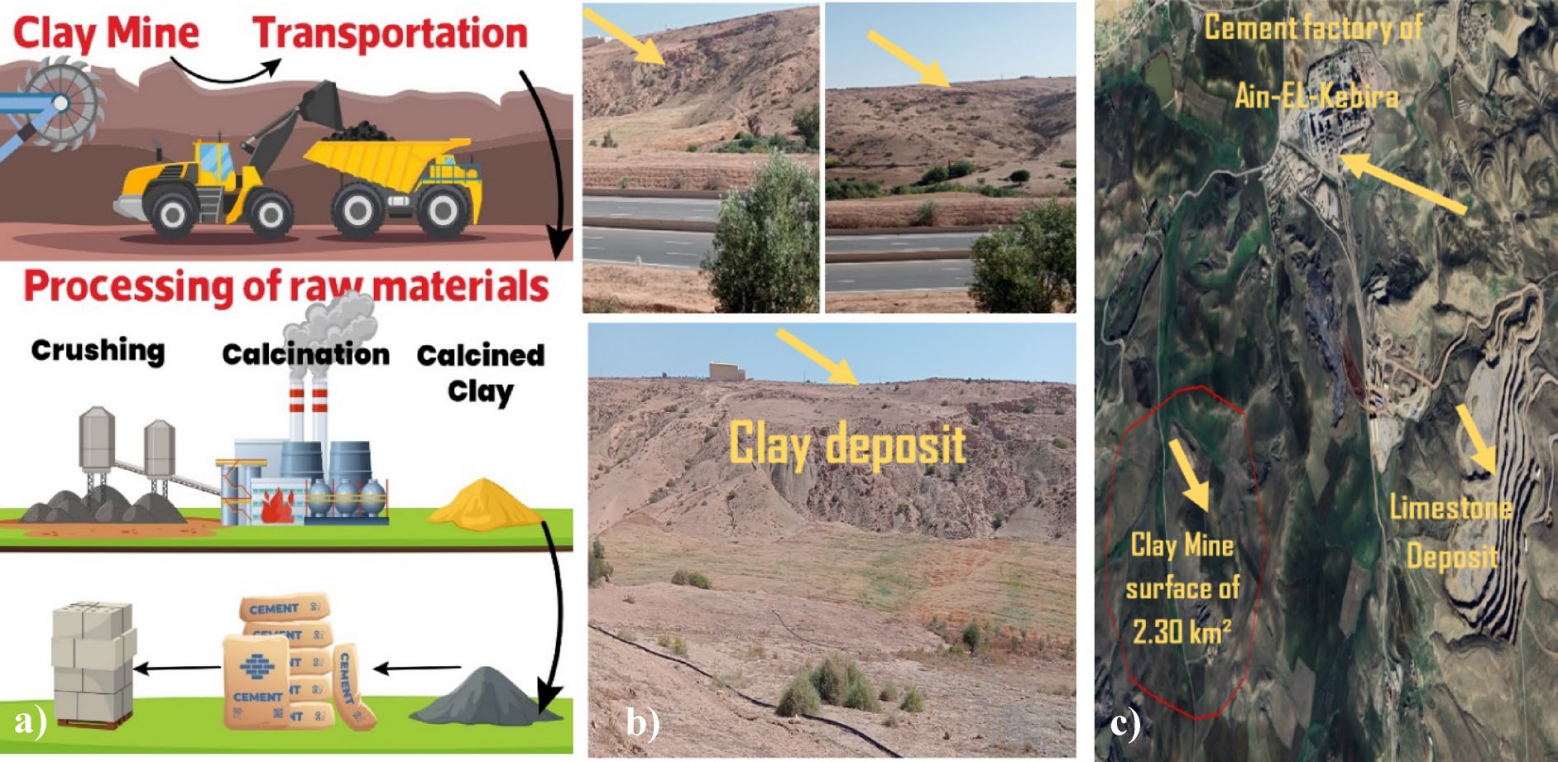
Clay mine: (**a**) process of producing clay, (**b**) clay deposit in Algeria, (**c**) location of clay deposit in relation to the cement company of Ain El-Kebira.

The silicon dioxide (SiO₂) and aluminum oxide (Al₂O₃) content in raw clay significantly influence both mechanical performance and thermal stability. Figure 2 presents the quantities of Si and Al used by researchers to assess the suitability of this clay as a supplementary cementitious material. SiO₂ content varies from 35 to 70%, while Al₂O₃ ranges from 5 to 45%, directly affecting pozzolanic reactivity and hydration product formation. A higher SiO₂ content enhances the generation of calcium silicate hydrate (C–S–H) gel, improving long-term strength. However, excessive SiO₂ (> 70%) may reduce reactivity if it is not well-balanced with Al₂O₃. Conversely, Al₂O₃ contributes to early-age strength development by forming calcium aluminate hydrates (C–A–H), but excessive levels (> 40%) can lead to the formation of unstable hydrates, in turn potentially compromising durability. The SiO₂/Al₂O₃ ratio, ideally between 2 and 4, ensures an optimal balance between reactivity and strength gain. Regarding thermal stability, SiO₂ remains stable up to 1000 °C, enhancing fire resistance, with Al₂O₃ influencing the dehydroxylation process. Kaolinitic clays typically undergo dehydroxylation between 500 and 800 °C, and result in highly reactive metakaolin. However, excessive Al₂O₃ may promote the formation of spinel or mullite phases (> 900 °C), thereby reducing pozzolanic reactivity. Therefore, maintaining an appropriate SiO₂/Al₂O₃ ratio is crucial for optimizing both mechanical properties and durability in cementitious applications.

This study introduces significant advancements by addressing critical gaps in research on cement additives, particularly in Algeria, where the reliance on limited materials, such as El Hadjar blast furnace slag and natural pozzolan, has constrained the development of sustainable alternatives. To enhance resource efficiency, this work explores the valorization of abundant local clay deposits as artificial pozzolans, offering a technically viable and environmentally sustainable solution for cement production. Beyond its pozzolanic reactivity, the use of calcined clay mitigates the environmental impact associated with raw clay accumulation, in turn transforming a potential waste material into a valuable supplementary cementitious component.

A key focus of this research is the case study of the Ain El Kebira cement plant, which currently sources natural pozzolan from a location 782 km away, leading to significant CO₂ emissions from transportation. By exploring the potential of locally available calcined clay, this study seeks to provide a sustainable alternative that optimizes logistical efficiency and reduces the carbon footprint of cement production. In addition to evaluating the mechanical performance of cement containing calcined clay, a comprehensive techno-environmental assessment was conducted, in which the impact on CO₂ emissions, resource efficiency, and economic feasibility was analysed. A novel multi-criteria evaluation framework was developed to holistically assess the sustainability of clay-based cementitious systems (Fig. 2). In order to achieve these objectives, compressive and flexural strength tests were systematically performed on cement mixtures with calcined clay substitution levels ranging from 10 to 30%. Particle fineness levels below 80 and 45 µm were considered. The findings not only confirm the technical feasibility of such substitutions, but also highlight their environmental and economic advantages, in turn supporting the transition towards more sustainable cement production. By integrating locally sourced calcined clay into industrial applications, this research provides a scalable model for reducing dependency on energy-intensive clinker, for minimizing impacts on the environmental, and for promoting eco-efficient construction materials in regions facing similar material constraints.

## Materials and methods

In this study, standard Ordinary Portland Cement (OPC) mortar mixtures comprising cement, sand, and water were prepared, where the cement to sand (C/S) and the water to binder (W/B) ratios were equal to 1/3 and 0.5, respectively. The used cement was an ordinary Portland cement (CEM I 42.5) provided by the Ain El Kebira Company (Sétif, Algeria), and was composed of 95% of clinker and 5% of gypsum in order to control the setting of cement. Table 2 presents the chemical and mineralogical characteristics of the used clinker, and shows that it complies with standard NF EN 197-1. This is due to the fact that the ratio of calcium oxide (CaO) to silica (SiO_2_) was equal to 3.11 (which is greater than 2). Moreover, the magnesium oxide (MgO) content was less than 5% by weight. The clinker mineralogical composition was calculated according to the Bogue empirical formula.

**Table 2:** Chemical and mineralogical properties of the used clinker.

Compound	SiO_2_	AlO_3_	Fe_2_O_3_	CaO	MgO	SO_3_	K_2_O	NaO
(%)	21.12	05.13	5.23	65.86	1.41	0.44	0.34	0.19
C_3_S = 65.6% C_2_S = 11.06% C_3_A = 4.74% C_4_AF = 15.91%			- Loss of Ignition L.O.I. = 0.28 according to NF EN 196–2 - Specific Surface Area S.S.A. = 3200 cm^2^/g - Density = 3220 kg/m^3^					

**Fig. 3:**
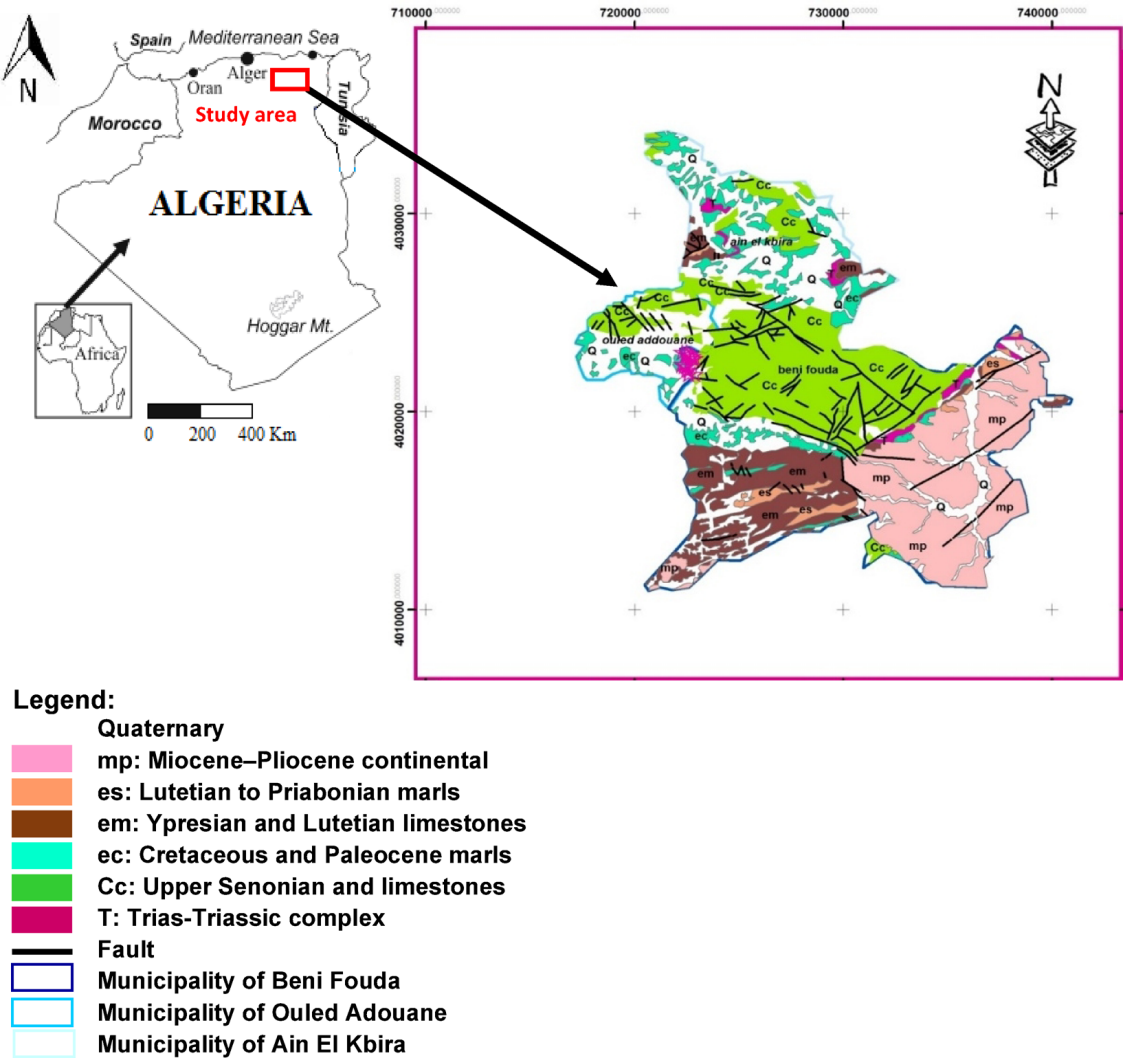
Geographic location and geological map of the clay deposit generated by par ArcGIS software version 10/www.arcgis.com (extracted from the geological map of Sétif, scale 1/200.000).

In the region of Theniet Mouloutou, the raw clay (RC) deposit (Figure 3) is located in the vicinity of national road No. 9, which connects Sétif to Ain El kebira. The deposit of interest belongs to the Cretaceous epoch formations, and is located about 3 km southwest of the cement company of Ain El kebira. It resembles a hill covering an area of 48 hectares, with a functional base width of 40 meters, distributed across two layers. The first layer (about 8 m) is composed of tender and very friable marly rocks. The second layer is composed of marl-clayey rocks. Both layers were interspersed by isolated beds of calcareous marl (10 cm) and limestone (3 to 6 cm).

The grain size distribution of the raw clay was performed by using laser diffraction analysis (LDA) (LA-960, Partica, HORIBA Scientific). The granulometric characteristics were also calculated. The Atterberg limits and methylene blue value (VBS) of the raw clay were performed according to NF P94-051 and NF P 94–068, respectively. The plasticity index of the raw clay was obtained by determining the liquid limit (LL) and plastic limit (PL). The chemical composition element was examined using X-Ray Fluorescence spectrometry (XRF) (Model no: ZSX Primus IV spectrometer). Fourier transform infrared (FTIR) measurements were performed with the use of an IRAffinity-1S spectrometer on a powdered solid sample mixed with 3% of the KBr that was prepared using the pressed disk technique. The measurements were conducted for a wave number range from 400 cm^−1^ to 4500 cm^−1^ and with 0.5 cm^−1^ of resolution. The mineralogical analysis used X-ray diffraction and was carried out on samples taken from Theniet Mouloutou using a Panalytical X Pert Pro diffractometer.

### Heat treatment

Calcination is a widely used treatment method developed to modify the properties of clay, aiming to enhance and broaden its potential applications. The pozzolanic reactivity of calcined clay is primarily attributed to the removal of structural water from its crystalline layers, resulting in an amorphous or semi-amorphous material with a high surface area and increased chemical reactivity^27^. Heat treatment destroys the crystal lattice, the water is eliminated, and silica and alumina are transformed into an amorphous state^44^. Therefore, it is necessary to study the behaviour of clay after exposure to high temperatures, and the influence of temperature on its microstructure. The thermal behaviour of clay was investigated for temperatures ranging from room temperature up to 1000 °C using a DSC-TGA instrument with a heating ratio of 10 °C/min. Thermogravimetric analysis (TGA) allows for the assessment of mass loss associated with phase changes as a function of temperature. Fourier transform infrared (FTIR) analyses were performed on heated samples at different temperatures ranging from 100 °C to 850 °C.

### Mortar preparation

Once the optimal calcination temperature was determined, the clay was heated at 700 °C and subsequently ground using a conventional ball mill. To optimize the particle size distribution and to evaluate the influence of fineness on the mechanical and environmental properties, the calcined clay was sieved into two granular fractions: < 45 µm and < 80 µm. This classification enabled the assessment of both fineness and substitution levels on the mechanical behaviour of mortars over time. Five cement replacement levels of 10%, 15%, 20%, 25%, and 30% by mass were considered (Table 3). For each fineness level, both reference (unmodified) and blended mortars were formulated in accordance with EN 196–1:2016. The fresh mixtures were cast into 40 × 40 × 160 mm^3^ molds, subjected to a 24-h curing period in a controlled humidity chamber, and then demolded and stored in water at 20 ± 1 °C until testing. A total of eleven distinct formulations were prepared, and mechanical performance was systematically evaluated at 2, 7, and 28 days.

**Table 3:** Mortar mixture proportions.

Calcined clay 45 µm or 80 µm (g)	Cement (g)	Sand (g)	Water (g)
0	450	1350	225
45	405		
67.5	382.5		
90	360		
112.5	337.5		
135	315		

## Results and discussion

### Clay characteristics

**Fig. 4:**
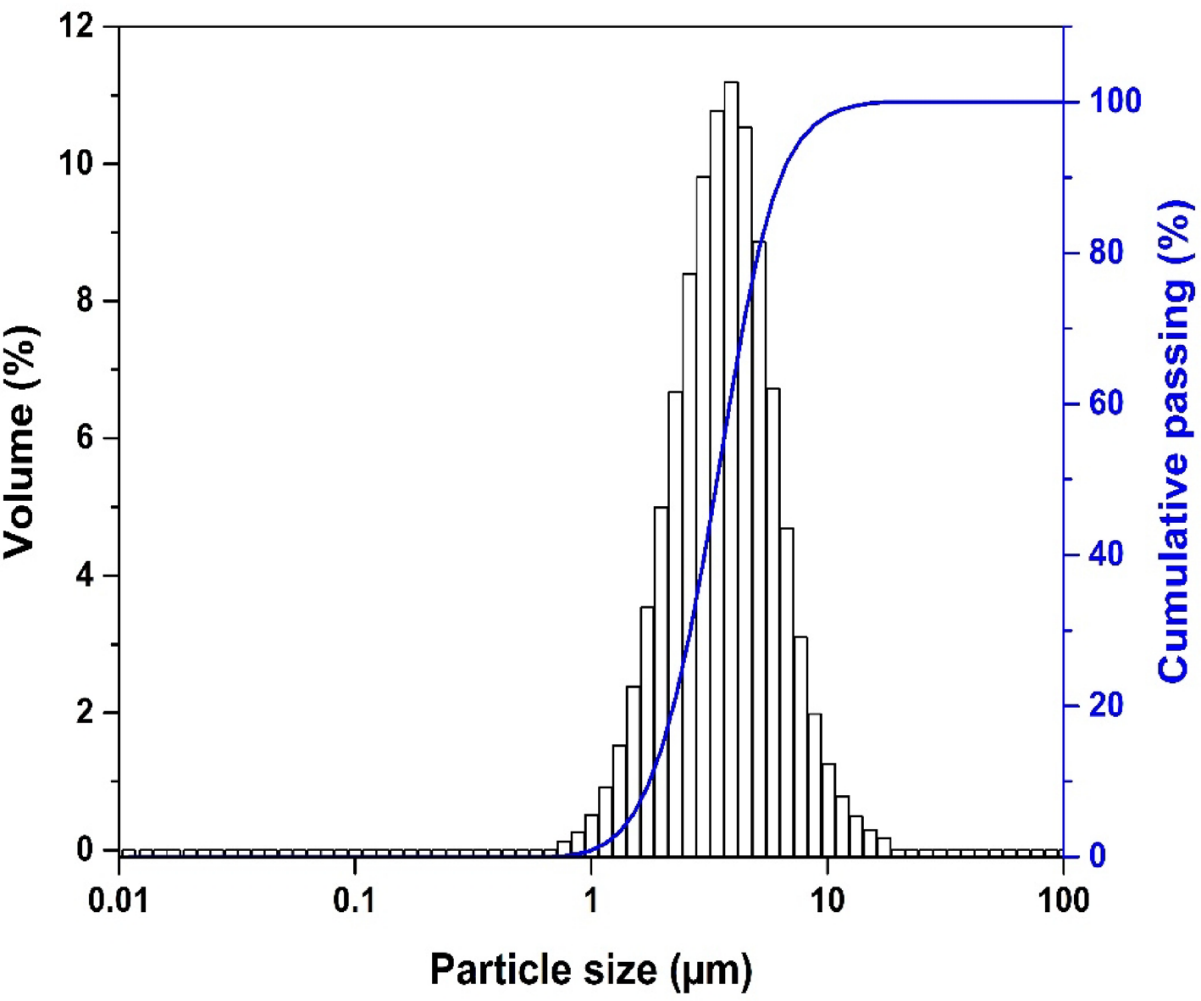
Particle size analysis of the raw clay.

The grain size distribution of the raw clay is shown in Fig. 4. Table 4 illustrates the granulometric and geotechnical characteristics of the raw clay. The clayey fraction (less than 2 µm) is of the order of 20% (it is of the order of 90% for a limiting diameter of 5 µm). The raw clay has a plasticity limit of 22.42. The plasticity index falls within the range of plasticity indices for kaolinite, as reported by Benredouane et al.^14^. These high values of plasticity were typical for clay materials and indicate that the sample in this study has good plasticity. Based on the relationships between geotechnical parameters and the content of phyllosilicates and amorphous materials established by Locat^45^, it was indicated that the studied clay contains less than 20% of phyllosilicates and amorphous phases.

**Table 4:** Physical properties of the tested raw clay.

	Soil properties	Standards	Values
Consistency limits	Liquid Limit (LL) (%)	NFP-94-051	44
	Plastic Limit (PL) (%)		22.4
	Plastic Index (PI) (%)		21.5
	Consistency Index (CI) (%)		1.7
	Methylene blue value (VBS)	NFP-94-068	5.01
Sieve analysis properties	D_10_	NFP-94-057	1.59
	D_30_		2.4
	D_50_		3.08
	D_60_		3.49
	D_90_		5.13
	Uniformity Coefficient (C_u_)		2.19
	Curvature Coefficient (C_c_)		1.03

Di: Grain diameter corresponding to i% passing (µm).

Flexural strength tests were performed on mortar specimens, with each reported value being the average of three measurements. After flexural testing, the compressive strength was determined using the two halves of the broken specimens, and the reported values represent the average of six half-specimens. All tests, including both flexural and compressive strength evaluations, were conducted in strict compliance with standard EN 196–1:2016 in order to ensure consistency and reliability of the results^46^.

The physicochemical characterization of the raw clay, as determined using X-Ray Fluorescence (XRF), Fourier-Transform Infrared Spectroscopy (FTIR), and X-Ray Diffraction (XRD), provides critical insights into its chemical composition, mineralogical structure, and functional properties. The XRF analysis, as shown in Table 5, indicates that the raw clay is mainly composed of SiO₂ (52.3%), Al₂O₃ (25.1%), and CaO (12.4%). The high SiO₂ and Al₂O₃ content suggests the presence of kaolinite (Al₂Si₂O₅(OH)₄), a phyllosilicate mineral commonly found in clay deposits. The elevated CaO content is indicative of calcite (CaCO₃), while the SiO₂/Al₂O₃ molar ratio of 2.09 confirms the presence of free quartz (SiO₂), as further supported by the XRD analysis. The combined percentage of minor oxides are in the order of 10.7%, which shows that the studied clay is not pure^38,47^. Moreover, the significant presence of Fe₂O₃ and K₂O suggests the coexistence of illite^37^, a non-expansive clay mineral. The loss on ignition of 17.6% reflects the high content of volatile components, primarily structural water and organic matter, which are characteristic of clay minerals.

**Table 5:** Chemical composition of raw clay (RC) and calcined clay (CC) at 700 °C, when compared to other clays.

Compound	SiO_2_	Al_2_O_3_	Fe_2_O_3_	CaO	MgO	SO_3_	K_2_O	Na_2_O	L.O.I
Raw clay (%)	37.5	17.9	7.01	14.9	1.45	0.75	1.03	0.46	17.6
Calcined clay (%)	44.8	20.9	7.01	18.3	1.74	1.71	1.29	0.58	-
Other popular clays									
	SiO_2_	Al_2_O_3_	Fe_2_O_3_	CaO	MgO	SO_3_	K_2_O	Na_2_O	L.O.I
Source clay (%)^36^	64.32	18.21	7.38	0.03	0.25	0.01	0.84	0.08	7.9
Calcine clay (%)^36^	68.49	19.82	8.18	0.04	0.24	0.02	0.96	0.1	1.09
Calcined marl (%)^34^	49.6	18.1	10.6	14.1	2.9	-	2.4	0.7	-
Iranian kaolins (%)^43^	53.38	43.18	1.29	0.05	0.35	-	1.11	-	0.52

**Fig. 5:**
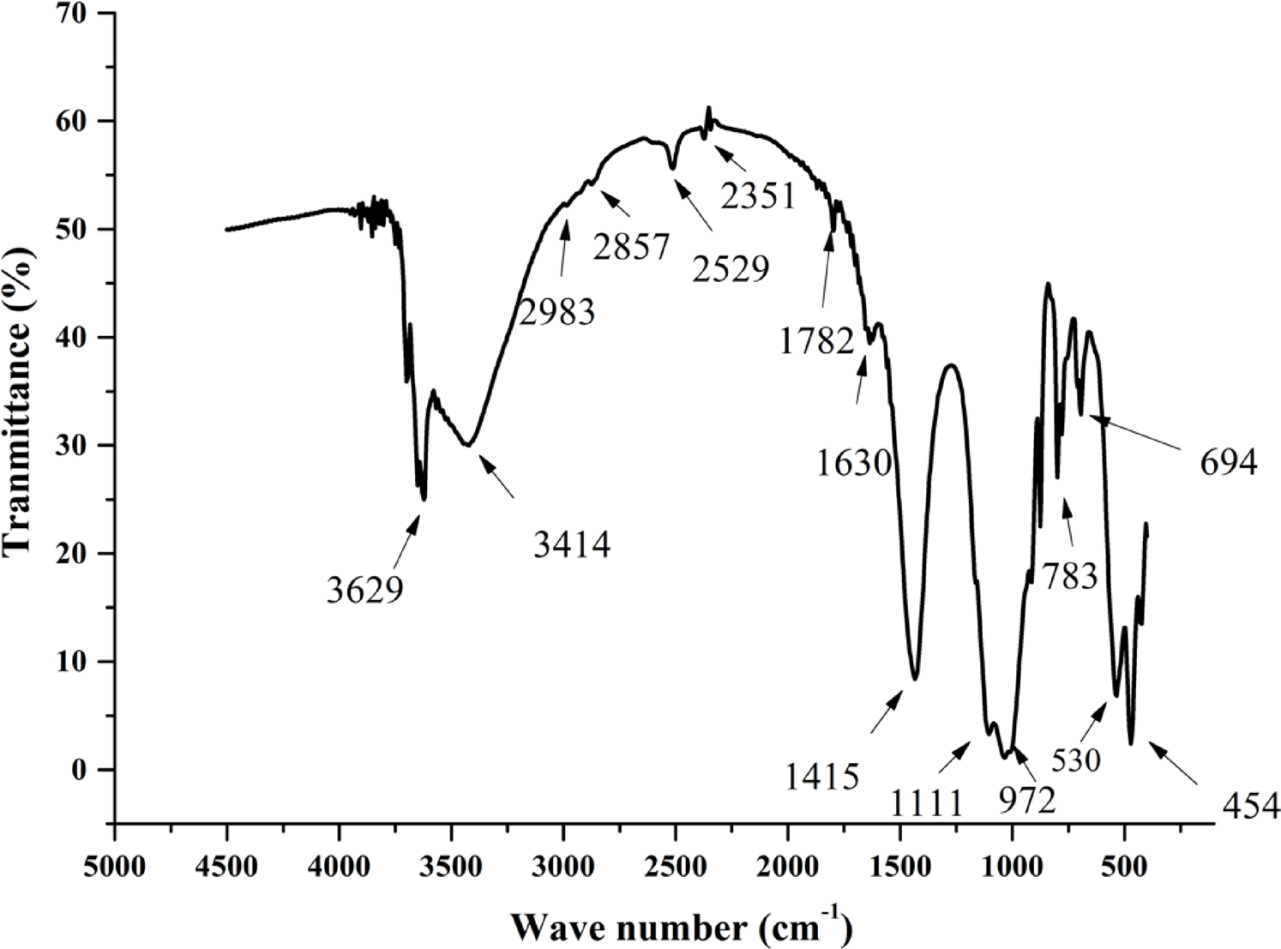
FTIR Analysis of the studied raw clay.

The FTIR spectroscopy was employed to elucidate the molecular structure and functional groups present in the raw clay. The FTIR spectrum (Fig. 5) exhibits distinct adsorption bands that correspond to kaolinite, quartz, and carbonates. The bands observed in the region of 3700–3620 cm⁻^1^, particularly at 3629 cm⁻^1^, are attributed to the stretching vibrations of the structural hydroxyl groups (–OH) in the kaolinite. The bands at 3414 cm⁻^1^ and 1630 cm⁻^1^ are associated with the elongation and deformation vibrations of adsorbed water molecules (H₂O), respectively. The presence of carbonates is confirmed by the characteristic bands at 875 cm⁻^1^ (out-of-plane bending of CO₃^2^⁻), 1436 cm⁻^1^ (asymmetric stretching of CO₃^2^⁻), and 2529 cm⁻^1^ (combination modes of CO₃^2^⁻). Additionally, the band at 2351 cm⁻^1^ corresponds to the elongation vibrations of carbon dioxide (CO₂) groups. The deformation vibrations of the Si–O bonds in the quartz are observed at 470 cm⁻^1^, 535 cm⁻^1^, 693 cm⁻^1^, and 796 cm⁻^1^, while the symmetric and asymmetric stretching vibrations of the Si–O–Si groups in the kaolinite and quartz are identified at 1032 cm⁻^1^. The bands at 998 cm⁻^1^ and 1110 cm⁻^1^ are attributed to the stretching vibrations of the Si–O bonds in the kaolinite, and the deformation vibration of the Al–OH bonds in the kaolinite is observed at 914 cm⁻^1^. Comparative analysis with other clays reported in the literature^17,34,36,37,43^ reveals that the studied clay conforms to the chemical requirements for Class N pozzolans as per ASTM C618^48^ , with SiO₂ + Al₂O₃ + Fe₂O₃ > 70% and SO₃ < 4%. However, the high CaO content (12.4%) distinguishes it from typical kaolinitic clays, aligning it more closely with marl, a calcareous clay. This similarity is further supported by the chemical composition of calcined marl, as reported in prior studies^36^. The increase in oxide percentages after calcination is attributed to the decomposition of carbonates and the removal of structural water, a phenomenon consistent with observations in the study of Danner et al.^34^.

**Fig. 6:**
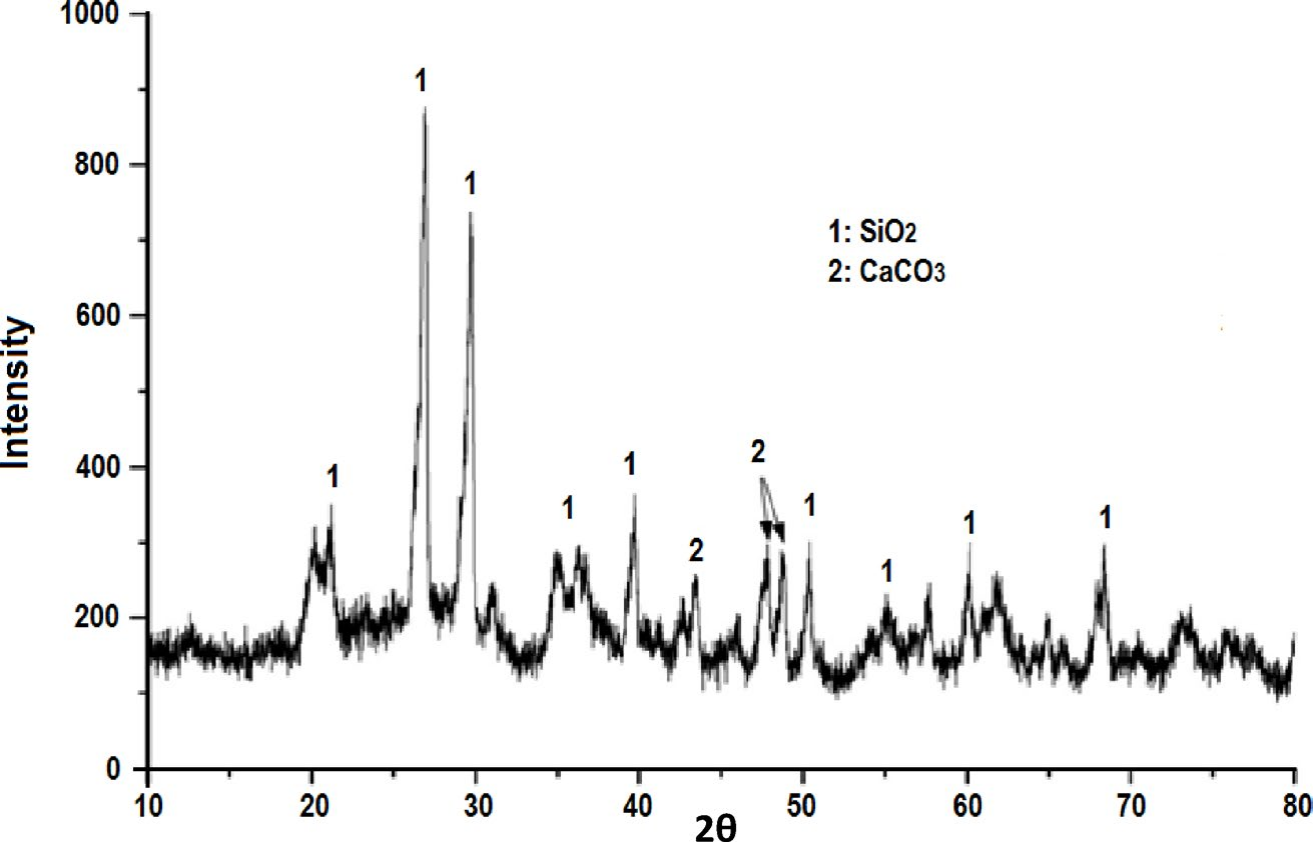
X-ray diffraction pattern of the raw clay.

The raw clay is composed of quartz (SiO_2_), calcite (CaCO_3_), illite [(K, H_3_O) Al_2_Si_3_, AlO_10_ (OH)_2_] and kaolinite (Al_2_Si_2_O_5_(OH)_4_). In the X-ray diffractogram illustrated in Fig. 6, the presence of two intense lines constituting the main clay phase can be noted. This corresponds to the presence of a mixture of quartz and calcite. These results show that this clay is heterogeneous, and confirm the previously obtained results from the X-ray fluorescence analysis. The X-ray diffractogram highlights the existence of different mineralogical phases: quartz, with diffraction peaks at 2θ angles of 26.29°, 39.45°, 50.11°, and 60.27°; calcite, at 2θ angles of 29.47°, 43.12°, and 68.43°; and kaolin, at 2θ angles of 11.95°, 20.50°, and 36.20°. Traces of illite are also observed, with diffraction peaks at 2θ angles of 31.52° and 36.20°.

**Fig. 7:**
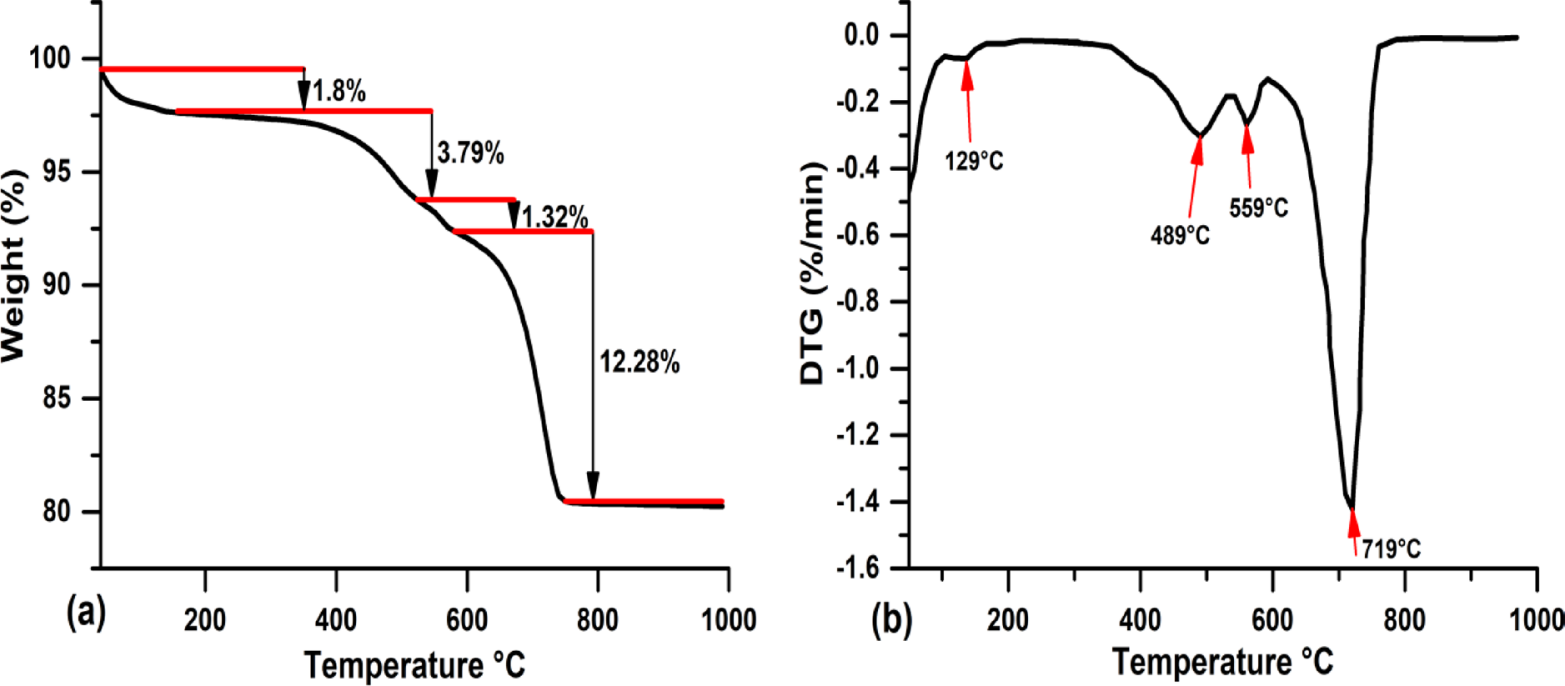
TGA and ATD curves for the raw clay.

Thermal analysis of the raw clay is illustrated in Fig. 7. As is shown on the TGA curve, a loss of weight is noted over several steps, with a maximum of 19.7% at 1000 °C. The first mass loss is observed at an endothermic peak around 129.40 °C, corresponding to a 1.8% mass loss, which is attributed to the release of chemically free water (dehydration) and volatile elements. This is followed by the release of chemically bound water (dihydroxylation) between 450 °C and 600 °C. The dihydroxylation peaks are notably significant, with mass losses of 3.79% and 1.32% attributed to the dihydroxylation of kaolinite and illite, respectively^49^. The two small peaks at 489.13 °C and 559.38 °C were linked to the presence of quartz. The ATD curve of this clay also shows a strong endothermic peak at 719.02 due to the dihydroxylation of a mineral from the smectite group, with a corresponding mass loss of 12.28%.

**Fig. 8:**
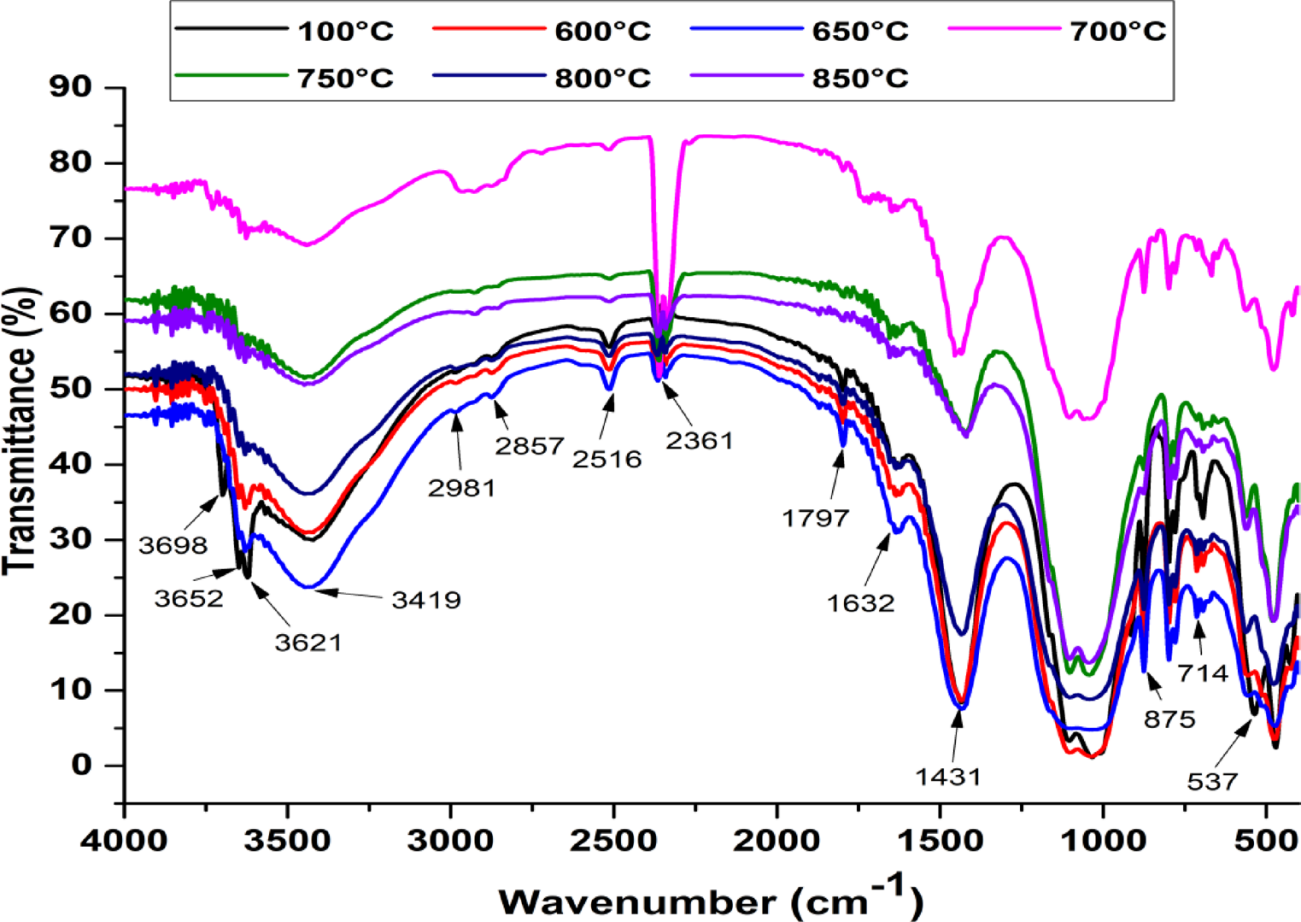
Infrared spectrum of calcined clay.

The results of the Fourier Transform Infrared Spectroscopy (FTIR) on the heated samples at different temperatures (from 100 to 850 °C) are presented in Fig. 8. The comparison between the infrared spectra by Fourier transform (FTIR) of geopolymers treated at room temperature up to 850 °C shows important differences. Indeed, on the FTIR spectra of materials treated from 25 °C to 650 °C, the bands at 3698, 3652, 3621, 3419 and 1630 cm^−1^ still appear, which is characteristic of kaolinite hydroxyl groups. This shows that for a heat treatment reaching 650 °C, the geopolymerization reaction is still incomplete within the material^50^. Thus, the comparison of the spectra of clay treated from 25 to 650 °C with those of the products obtained after treatment at temperatures of 700 to 850 °C shows that the latter no longer contain kaolinite (a mineral precursor of synthesis). This is confirmed by the sharp decrease or absence of absorption bands at 2529, 1431 and 875 cm^−1^, which is attributed to Kaolinite (CaCO_3_) stretching vibrations. It was observed that the band at 914 cm⁻^1^, corresponding to the deformation vibrations of the Al–OH bond in kaolinite, disappeared. Similarly, the band at 535 cm⁻^1^, associated with the symmetrical elongation vibrations of the Si–O-Si and Al–O–Si bonds in kaolinite, also vanished. A sharp decrease in the intensity of absorption bands of organic matter can also be observed, more evidence of the elimination of kaolinite during the heat treatment. On the other hand, the absorption band around 2351 cm^−1^, originating from the carbon dioxide largely dissolved in the samples rich in lime^51^, increased in intensity for the clay treated at 700 °C only. This can be reflected in the enrichment of the material in CaO due to the partial decomposition of calcium carbonates CaCO_3_ into CaO. This band returns to its initial state for the clays treated at temperatures above 750 °C, reflecting the absence of CaO transformed into Ca(OH)_2_ groups by its hydration at atmospheric temperature under ambient conditions due to the hygroscopic property of CaO^50^. The infrared study confirmed the presence of kaolinite in the clay. Quartz, carbonates and traces of organic matter were also detected in our clay.

**Fig. 9:**
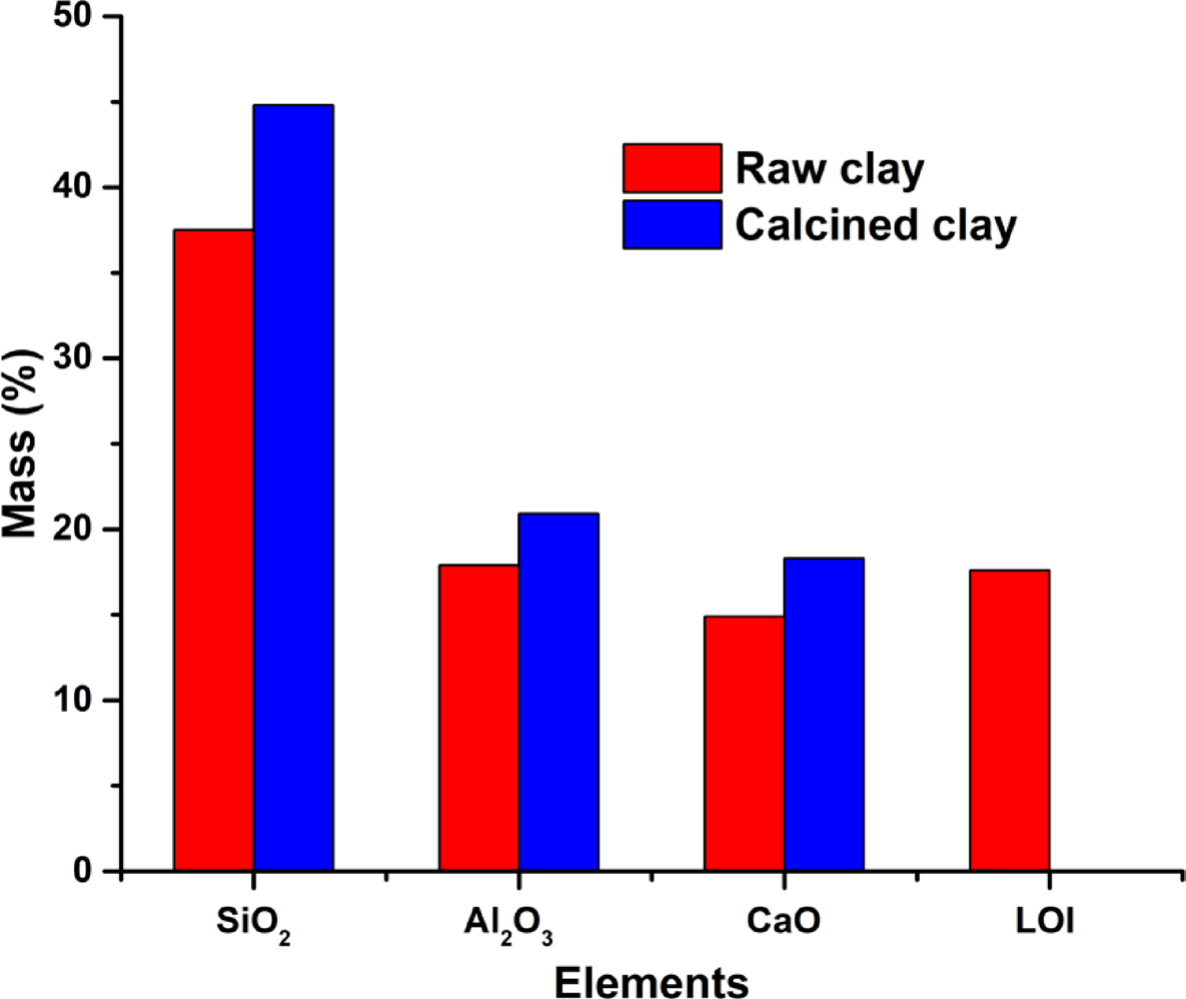
Principal element of the clay before and after calcination at 700 °C.

The heat treatment of the clay induced the geopolymerization reaction at approximately 700 °C, which facilitated the activation of the clay by causing the disappearance of kaolinite’s characteristic reflections above this temperature. Based on the TGA and FTIR analyses, 700 °C was determined as the optimal temperature for the heat treatment. After grinding the clay to a particle size of less than 80 µm, it was subjected to heat treatment at 700 °C using a Nabertherm electric furnace with a maximum temperature capacity of 3000 °C. The heat treatment cycle (TTh) consists of a temperature ramp up to a speed of 5 °C/min, followed by an isothermal bearing at the maximum temperature 700 °C with a hold of 3 h. The chemical composition of the calcined clay is reported in Table 5. The ratio of oxides increased after the calcination of the clay (Fig. 9). This phenomenon is due to the loss of hydroxyl groups (OH) after material heating, thus allowing the formation of new bonds (Si–O, Al-O, and Ca-O) and therefore an increase in oxides levels. Moreover, the loss on ignition is reduced due to the degradation of organic molecules and the evaporation of water after heating at high temperatures.

1 $${\text{Si}} - {\text{OH}} + {\text{Si}} - {\text{OH}} \to {\text{Si}} - {\text{O}} - {\text{Si}} + {\text{H}} - {\text{O}} - {\text{H}}$$

### Compressive strength

**Fig. 10:**
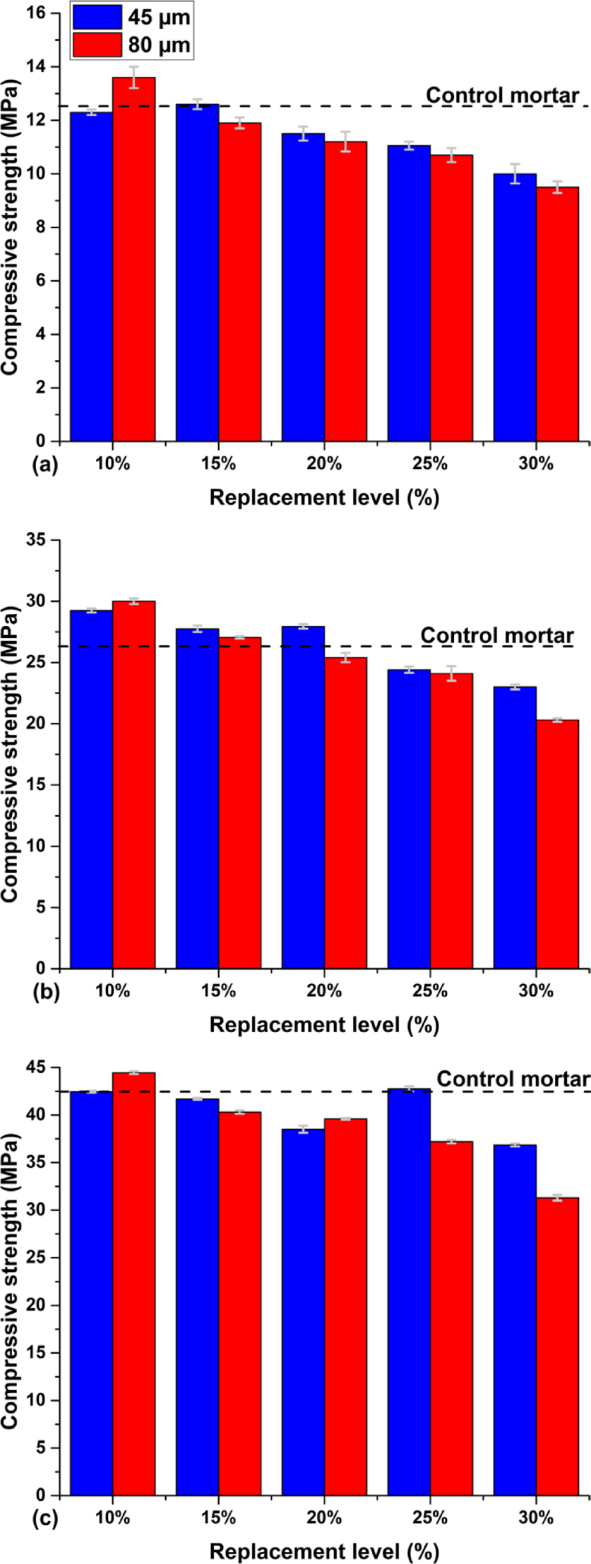
Evolution of the compressive strength index: (**a**) at 2 days, (**b**) at 7 days, and (**c**) at 28 days.

The pozzolanic activity of the clay was evaluated with regards to compressive strength on normal mortars at 2, 7, and 28 days. Figure 10 shows the evolution of the mortars’ strength as a function of the replacement ratio of 10%, 15%, 20%, 25%, and 30% for different finenesses (45 µm and 80 µm) at 2, 7 and 28 days of hydration.

The evolution of compressive strength in cementitious mixtures incorporating calcined clay as a partial cement substitute is governed by the progressive hydration of cementitious materials and the interaction between cement and mineral additions. At early ages, the compressive strength of mixtures containing calcined clay does not surpass that of the control mortar. This phenomenon can be attributed to the dominant influence of the primary hydration reactions of clinker minerals, such as alite (C₃S) and belite (C₂S), which primarily dictate early-age strength development. Calcined clay, as a pozzolanic material, relies on the availability of portlandite (Ca(OH)₂), a byproduct of these primary hydration reactions, to initiate its own pozzolanic activity. Consequently, its contribution to early-age strength remains limited due to the delayed onset of the pozzolanic reaction. By 7 days of hydration, mixtures with calcined clay substitution levels below 15% exhibit compressive strengths exceeding those of the control mortar, irrespective of the fineness of the calcined clay. This enhancement can be explained by the physico-chemical effects of the mineral addition^8,35,44^. As reported in a prior study^52^, calcined clay particles act as nucleation sites for the precipitation of hydration products, facilitating a more uniform and compact distribution of calcium silicate hydrate (C–S–H) gels within the cement matrix. This microstructural refinement leads to improved packing density and enhanced mechanical strength, which is consistent with findings from earlier research. At 28 days of hydration, all mixtures achieve compressive strengths exceeding 75% of the control mortar, regardless of the substitution ratio or fineness of the calcined clay. This result is primarily driven by the pozzolanic reaction between the amorphous aluminosilicates present in calcined clay and the portlandite released during cement hydration. The pozzolanic reaction generates secondary C–S–H, which is denser and more stable than the primary hydration products formed at early ages. The delayed nature of this reaction accounts for the gradual strength gain observed over time^53^.

The kinetics of cement hydration and the pozzolanic reaction differ significantly. Cement hydration proceeds rapidly during the initial days, producing portlandite and primary C–S–H, which in turn contribute to early-age strength^54^. In contrast, the pozzolanic reaction progresses more slowly, becoming increasingly significant as portlandite accumulates and the amorphous phases in calcined clay react to form additional C–S–H. This interplay between the two reactions results in the long-term enhancement of mechanical properties^55^. Although hydration begins immediately upon the mixing of water and cement, the full chemical contribution of pozzolanic materials, such as calcined clay, occurs only after sufficient secondary C–S–H has formed. This highlights the importance of both the quantity and fineness of calcined clay in optimizing its reactivity and long-term performance. Furthermore, the continued pozzolanic reaction contributes to the gradual refinement of the pore structure, enhancing not only mechanical strength but also the durability of the material^56^. These findings underscore the critical role of calcined clay in improving the microstructural and mechanical properties of cementitious systems over time.

Belkadi et al.^57^ investigated the use of glass powder and crushed concrete as cement substitutes. The obtained results highlight differences in reactivity, adhesion properties, and mechanical performance, while at the same time emphasizing the impact of substitution rates on the mechanical properties of mortars. In Belkadi et al.'s study^57^, the glass powder exhibited an optimal performance at a 10% substitution level, with a compressive strength of 54.24 MPa. However, a progressive decrease in strength was observed with increasing substitution rates, with strengths of 45.62 MPa at 15% and 43.99 MPa at 20%. This trend can be attributed to the initial enhancement of the pozzolanic reaction at low substitution levels, followed by a saturation of this reaction at higher dosages, which limits the formation of solid reaction products. The role of particle fineness in activating the pozzolanic reaction is crucial, and the observed reduction in strength at higher substitution rates is linked to the limited reactivity of glass powder at elevated ratios, resulting in a less effective pozzolanic activity and, consequently, a decline in mechanical properties^58,59^. Similarly, the crushed concrete in Belkadi et al.'s study^57^ showed comparable results, with a compressive strength of 54.24 MPa at 10% substitution. This decreased to 45.62 MPa at 15% and 43.99 MPa at 20%. Crushed concrete, being composed of mineral aggregates and fine concrete particles, benefited from its higher density and better mechanical characteristics at low substitution rates. However, a reduction in performance was observed beyond 10%, which can be attributed to the less efficient interaction between crushed concrete particles and cement, in turn leading to a diminished adhesion and densification of the hydration products. This decrease in performance at higher substitution rates underscores the limited reactivity of crushed concrete, which results in a decrease in the mechanical properties of cement, especially at higher substitution levels. In contrast, the results for calcined clay in this study, at both 45 µm and 80 µm fineness, demonstrate a different trend. The calcined clay at 45 µm exhibited a compressive strength of 42.45 MPa at 10% substitution, which decreased to 41.7 MPa at 15% and 38.5 MPa at 20%. The relatively lower strength observed for calcined clay compared to glass powder and crushed concrete can be attributed to the inherently lower pozzolanic reactivity of calcined clay, especially at higher substitution rates. Furthermore, the lack of sufficient reactive components at these higher substitution levels decreases the mechanical performance of the mortars. However, fineness plays a key role in the performance: calcined clay at 80 µm provides a higher strength of 44.45 MPa at 10% substitution, but, like the 45 µm calcined clay, the performance rapidly declines with increasing substitution rates, reaching 40.3 MPa at 15% and 39.6 MPa at 20%. This progressive decrease in strength of calcined clay at higher substitution levels can be explained by several factors, namely: (1) the lower reactivity of calcined clay when compared to other more reactive pozzolanic materials (such as glass powder and crushed concrete^57^ limits the formation of reaction products that in turn contribute to enhancing the matrix strength; (2) The dilution of cement hydration products with increasing amounts of calcined clay, along with the influence of fineness leading to the agglomeration of calcined clay particles, necessitates the addition of more water^60,61^, As a result, the strength decreases, accompanied by a reduction in both the density and cohesion of the mortar . Despite substituting 10% of the cement with activated calcined clay, the mechanical resistance of the mixture remains closer to the guaranteed cement strength at 28 days (CEM I 42.5). This is considered an advantageous gain in limiting CO_2_ emissions and the use of natural resources. Our results are in good agreement with those of Belkadi et al.^57^.

Despite the partial substitution of 10% of the cement with activated calcined clay, the mechanical strength of the mixture remained closer to the 28-day compressive strength guaranteed by the reference cement (CEM I 42.5). This outcome represents a significant advantage in terms of sustainability, as it not only reduces CO₂ emissions associated with cement production, but also decreases the consumption of non-renewable natural resources. Such findings underscore the potential of incorporating supplementary cementitious materials to achieve environmentally favourable concrete mixtures without compromising performance.

### Flexural strength

**Fig. 11:**
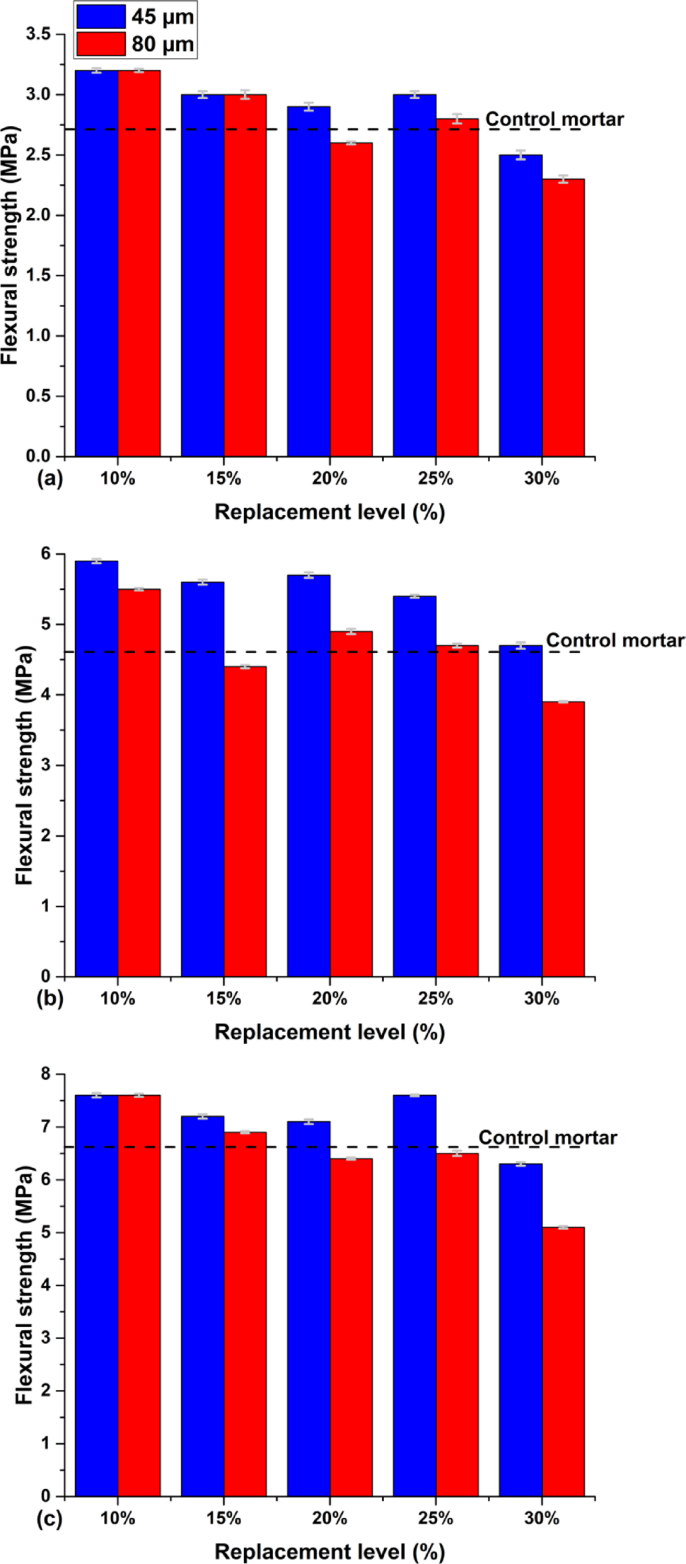
Evolution of the flexural strength index: (**a**) at 2 days, (**b**) at 7 days, and (**c**) at 28 days.

Figure 11 shows the flexural strength evolution of the mortars as a function of the replacement ratios (10%,15%, 20%, 25% and 30%) for different finenesses (45 µm and 80 µm) at 2, 7 and 28 days of hydration.

The flexural strength test results reveal a trend similar to those of compressive strength, but with a less significant reduction. Notably, the mortars incorporating the calcined clay generally surpass the control mortar in flexural strength, achieving increases of up to 120% at 7 days. The influence of fineness is evident, as finer calcined clay (45 µm) consistently outperforms coarser clay (80 µm). For instance, at 7 days and a 20% replacement, the 45 µm variant achieved 5.7 MPa, exceeding the 80 µm variant’s 4.9 MPa by approximately 16.3%. At 28 days, this trend persists, with the 45 µm clay at a 30% replacement reaching 6.3 MPa, compared to 5.1 MPa for 80 µm, marking a 23.5% improvement. Despite the advantages of finer particles, higher substitution rates (25–30%) tend to reduce strength when compared to lower rates (10–20%). This is due to the reduced clinker content, which in turn limits hydration. These findings confirm the significant influence of the calcined clay’s fineness and replacement ratio on flexural strength, and aligns with the results of Ghrici et al.^35^, who also demonstrated that incorporating calcined clay induces substantial improvements in the mechanical properties of cementitious materials.

### Mechanical and economic analysis

To evaluate the mechanical performance of mortars, this study investigated cement substitution ratios ranging from 10 to 30%, using particles with fineness levels of less than 40 μm and 80 μm. The mechanical performance ratio was calculated by comparing the compressive strengths of the modified mortars to those of the reference mortars. This analysis determined the precise impact of substitution and particle fineness on the mechanical properties of the mortars, thereby providing insights for optimizing mortar formulations based on structural requirements. To compare these results, the following mechanical performance ratio was used^62,63^:

2 $$\begin{array}{c}MPR=\frac{4\times \frac{{F}_{cm,mixes}}{{F}_{cm,ref}}+2\times \frac{{F}_{fm,mixes}}{{F}_{fm,ref}}}{6}\times 100 \left[\%\right]\end{array}$$

where *F*_*cm, mixes*_ is the compressive strength of the studied mortar, f_cm, ref_ represents the compressive strength of the reference mortar, F_*fm*_, _mixes_ is the flexural strength of the studied mortar, and *F*_*fm, ref*_ represents the flexural strength of the reference mortar.

In order to compare the results of the mechanical performance ratio (MPR), ΔMPR (delta MPR) was used in the following manner:

3 $$\begin{array}{c}\Delta MPR=\frac{{\text{MPR}}_{Series}}{{\text{MPR}}_{Reference}}\end{array}$$

The obtained results are presented in Table 6.

**Table 6:** Mechanical properties of the studied cementitious mortars.

Series	MPR_28 days_ (%)	ΔMPR_28 days_
MR	100	1
M10-45	104.97	1.05
M15-45	101.78	1.02
M20-45	96.25	0.96
M25-45	105.44	1.05
M30-45	89.62	0.9
M10-80	108.11	1.08
M15-80	98.06	0.98
M20-80	94.44	0.94
M25-80	91.18	0.91
M30-80	74.86	0.75

**Fig. 12:**
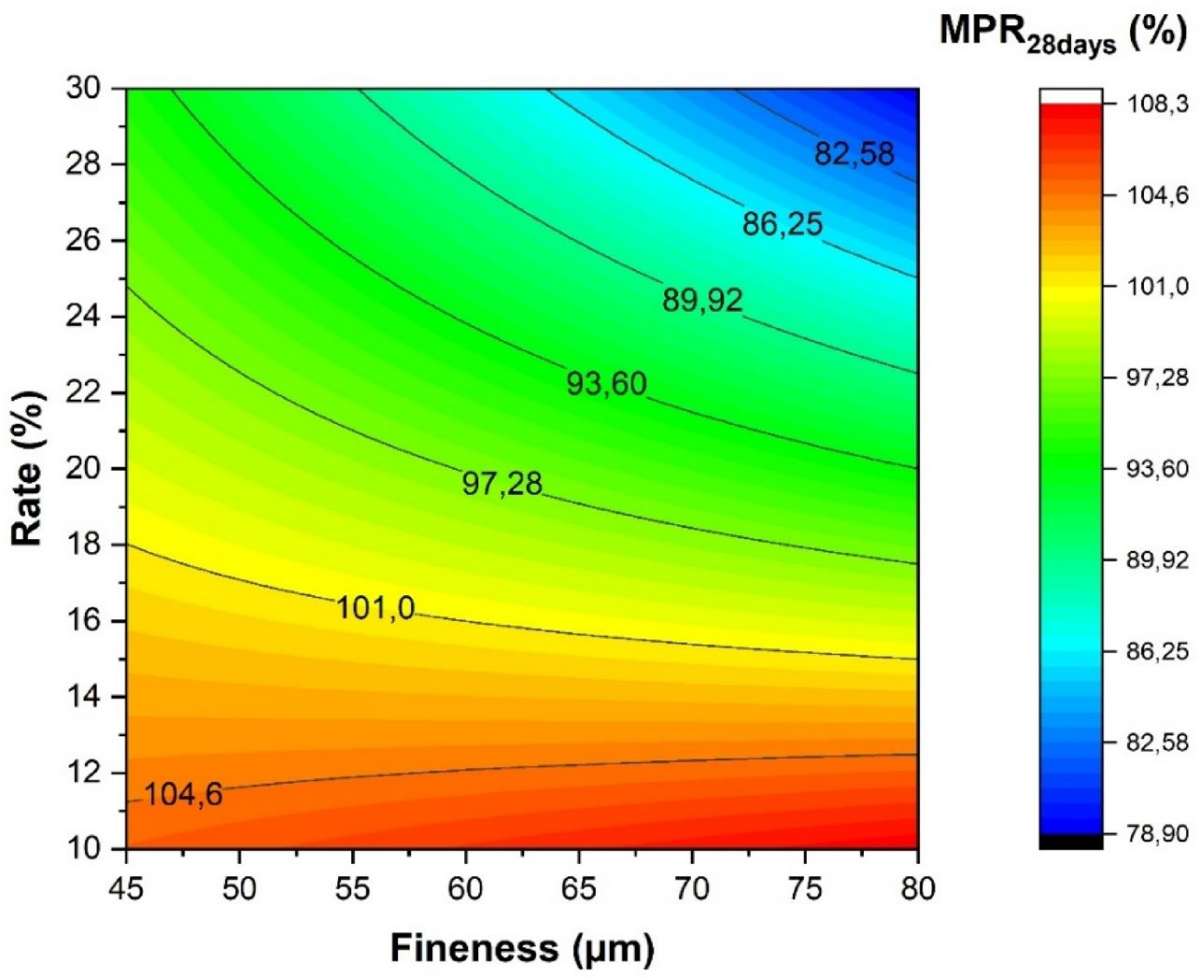
Iso-response of the mechanical performance ratio.

The evolution of the mechanical performance ratios as a function of the substitution ratio and the fineness of the addition is shown in Fig. 12. The results obtained demonstrate that both parameters have a significant influence on the mechanical response.

When finer fillers are used, the mechanical performance increases. This can be attributed to the filling effect, where the smaller particle size of the fine fillers allows them to occupy the voids within the cement matrix more effectively. This results in a denser and more compact microstructure, which enhances the overall strength and durability of the material. The improved packing density reduces the porosity and increases the resistance to cracking and other mechanical stresses. On the other hand, decreasing the substitution ratio also positively impacts mechanical performance. A lower substitution ratio means that a higher proportion of cement is retained in the mix, which in turn increases the amount of calcium silicate hydrate (C–S–H) produced during the hydration process.

The calculation of energy required for cement production encompasses the manufacturing process, which includes the thermal treatment of raw cement powder and the final grinding of the clinker^64^. The required energy (E, expressed in kWh/t of binder) can be determined using the following equation:

4 $$\begin{array}{c}E=C\times \left({E}_{pc}+{E}_{gc}\right)+\left(CP\times \left({E}_{pcp}+{E}_{gcp}\right)\right)\end{array}$$

where E is the amount of energy required to produce one ton of cement, expressed in kilowatt-hours (kWh/t); C and CP represent the relative proportions of the cement and clay powder used in the cement; and Epc, Egc, Epcp and Egcp denote the energy consumption (in kWh/t) of the thermal treatment process of the raw cement, the grinding of raw cement, the thermal treatment process of clay, and the grinding of clay, respectively.

According to previous research^65,66^, the values of energy for the production of one ton of binder typically range between 800 and 1200 kWh/t. The required energy to calcine one ton of clay is estimated to be 630 kWh/t^67^. Additionally, the energy necessary to achieve a fineness comparable to that of cement is estimated to be 8.4 kWh/t^68^. Table7 presents the obtained environmental results. In Algeria, electricity production primarily relies on natural gas, a relatively clean energy source. The generation of one kWh of electricity emits 0.55 kg of CO_2_^69^. Moreover, the cost of producing one kWh of electricity is estimated at 0.03 euros^57^. The obtained results for grinding energy, the CO_2_ emissions, and the cost of grinding energy are summarized in Table 7.

**Table 7:** Environmental and cost results.

Series	Energy (kwh/t)	CO_2_ (kg)	Cost (Euro)
Reference	1000	550	30
M10-45	964.68	530.57	28.94
M15-45	947.02	520.87	28.41
M20-45	929.36	511.15	27.88
M25-45	911.7	501.44	27.35
M30-45	894.04	491.72	26.82
M10-80	963.84	530.11	28.92
M15-80	945.76	520.17	28.37
M20-80	927.68	510.22	27.83
M25-80	909.6	500.28	27.29
M30-80	891.52	490.34	26.75

The obtained results indicate that substituting cement with calcined clay results in a reduction in grinding energy, CO₂ emissions, and associated grinding energy costs.An increase in the substitution ratio results in a decrease in the overall energy consumption, whereas increasing the fineness of the material leads to higher grinding energy requirements. Despite these increases in fineness, the addition of calcined clay has proven to be effective when compared to the reference cement.

Belkadi et al.^57^ conducted a techno-environmental study on the partial replacement of cement with glass powder. Their results showed that substituting cement with 10%, 15%, and 20% of glass required production energy values of 902, 853, and 804 kWh per ton. respectively. In contrast, this study indicates that substituting cement with 10%, 15%, and 20% of calcined clay requires a grinding energy of 964.68, 947.02 and 929.36 kWh per ton, respectively. The results of the siliceous sands analyzed in Hebbache et al.'s study^70^, with finenesses of 459, 497, and 543 m^2^/kg, only required only grinding energy that ranged from 807.46 kJ/kg to 929.36 kJ/kg. The non-calcination in sand processing results in significantly lower energy requirements compared to the calcination of clay. The higher energy demand for calcined clay, primarily due to the calcination process at 700–800 °C, raises concerns about its energy efficiency when compared to glass powder, which requires minimal processing. This energy intensity can increase indirect CO_2_ emissions, particularly if fossil fuels are used, potentially offsetting some of the environmental benefits of reduced clinker usage. However, calcined clay significantly lowers direct CO_2_ emissions in cement production, contributing to a net reduction in greenhouse gases. The sustainability of calcined clay can be enhanced by integrating renewable energy sources for calcination and adopting energy-efficient technologies. In contrast, glass powder, while less energy-intensive, is limited by the availability of waste glass, making it less scalable.

The results highlight the significance of optimizing the substitution ratio and fineness to achieve the best balance between energy efficiency and mechanical performance. By carefully selecting the proportion and fineness of the substituted material, it is possible to enhance the overall sustainability of cement production.

**Fig. 13:**
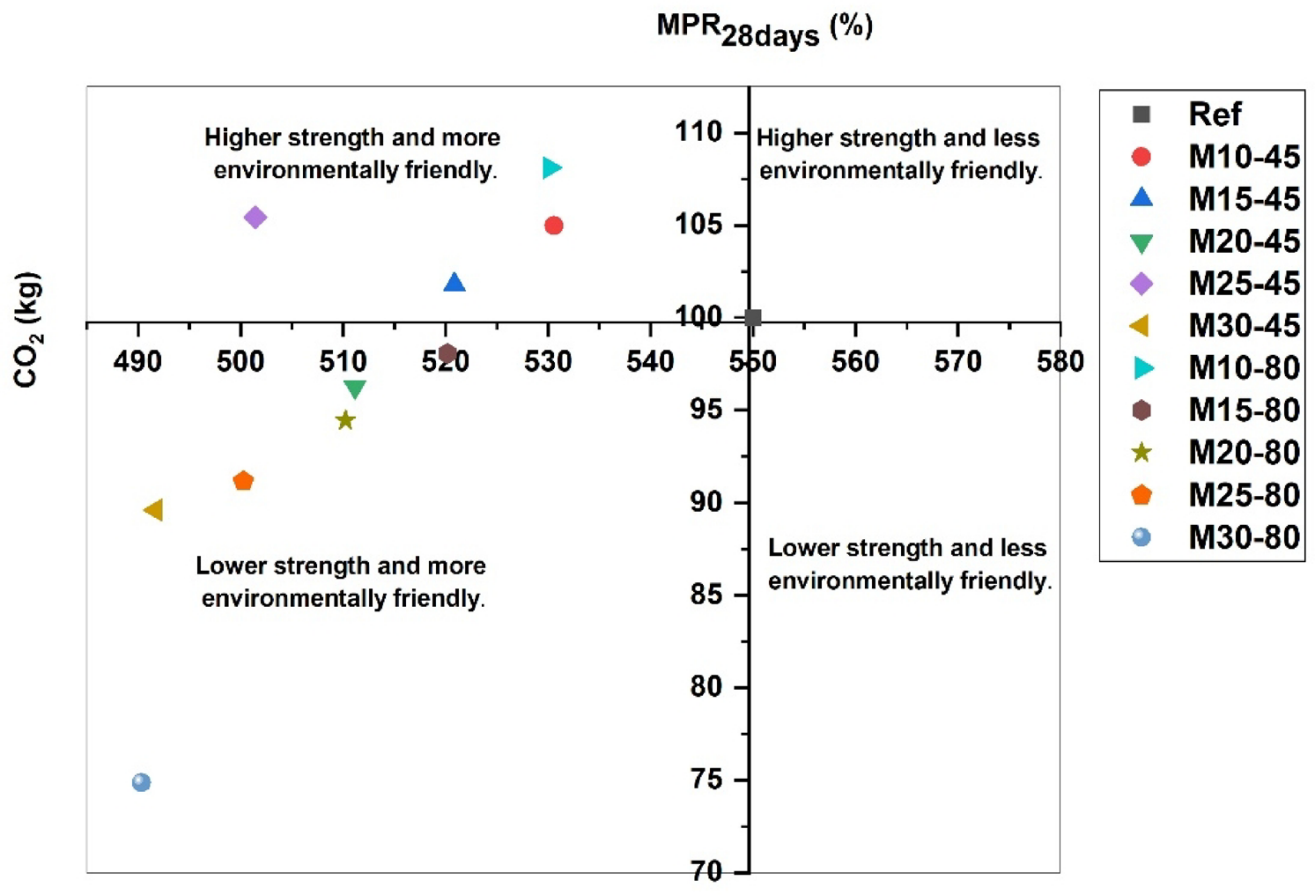
Mechanical performance ratio (MPR) versus CO_2_ emission for cement mortars.

Figure 13 presents a combined analysis of mechanical performance and environmental impact. The results indicate that substituting 10%, 15%, and 25% of cement with calcined clay that has a fineness below 45 µm produces more efficient and environmentally friendly mortars. Additionally, even a 10% substitution with calcined clay with a fineness below 80 µm yields similar benefits. In contrast, the other mixtures result in less efficient mortars, but more environmentally friendly mortars. The substitution of cement with calcined clay not only reduces the carbon footprint of concrete by lowering the clinker content—thus decreasing CO₂ emissions associated with its production—but also improves the sustainability of concrete in service. The durability of cementitious materials incorporating calcined clay is closely linked to microstructural changes that occur due to pozzolanic reactions and filler effects^61^. When compared to Portland cement, the partial replacement of clinker with calcined clay refines the pore structure by promoting additional C–S–H and C–A–S–H gel formation while at the same time consuming portlandite (CH), thereby reducing the volume of large capillary pores and enhancing resistance to aggressive environments^71^. The carbonation depth in mortars containing calcined clay is influenced by the reduction in the portlandite content and the densification of the microstructure. While a lower CH content may increase carbonation susceptibility at early ages, the improved packing density and refined pore network mitigate CO₂ penetration, in turn stabilizing long-term carbonation resistance^72^. Similarly, sulfate resistance is enhanced due to the lower availability of CH, which limits the formation of expansive ettringite and gypsum in sulfate-rich environments. The presence of alumina in calcined clay contributes to the formation of stable monosulfate phases, further reducing sulfate-induced deterioration. Permeability is significantly reduced as the finer particle size and secondary pozzolanic reactions refine the pore structure, thereby decreasing water absorption and ion diffusion. This densification translates into improved resistance against chloride ingress, reducing the risk of reinforcement corrosion in reinforced concrete applications. Consequently, the long-term structural performance of mortars incorporating calcined clay benefits from these durability enhancements, ensuring better resistance to environmental degradation while also maintaining mechanical integrity over extended service life^73^.

From a technical perspective, calcined clay has the potential to be used as a primary component in cement production (Table 8). Various types of cement can be formulated based on the mechanical and compositional requirements specified in EN 197–1. The current study demonstrates that it is feasible to produce CEM II/A-M 42.5 N using the M10-45 mixture, CEM II/A-M 32.5 R using mixtures M15-45, M15-80, M20-45, and M20-80, and CEM II/B-M 32.5 R using mixtures M30-45 and M25-80. However, there is potential for the further development and production of other cement types.

**Table 8:** Potential cement types based on EN197-1 requirements and study findings.

EN 197–1 composition requirements			The present study combinations satisfying the mechanical requirements		EN 197–1 Mechanical requirements		Calcined clay content satisfying the mechanical and composition requirements (%)
Strength class	CS (MPa)		Cement	CC content (%)	Cement type	Composition (%)	
	2 days	28 days					
32.5 N	≥ 16 at 7 d	≥ 32.5	M30-80	30	CEM II/B-M	21–35	30
32.5 R	≥ 10	≥ 32.5	M15-45	15	CEM II/A-M	06–10	15
			M15-80	15	CEM II/A-M	06–10	15
			M20-45	20	CEM II/A-M	06–10	20
			M20-80	20	CEM II/A-M	06–10	20
			M30-45	30	CEM II/B-M	21–35	30
			M25-80	25	CEM II/B-M	21–35	25
42.5 N	≥ 10	≥ 42.5	M10-45	10	CEM II/A-M	06–10	10

**Fig. 14:**
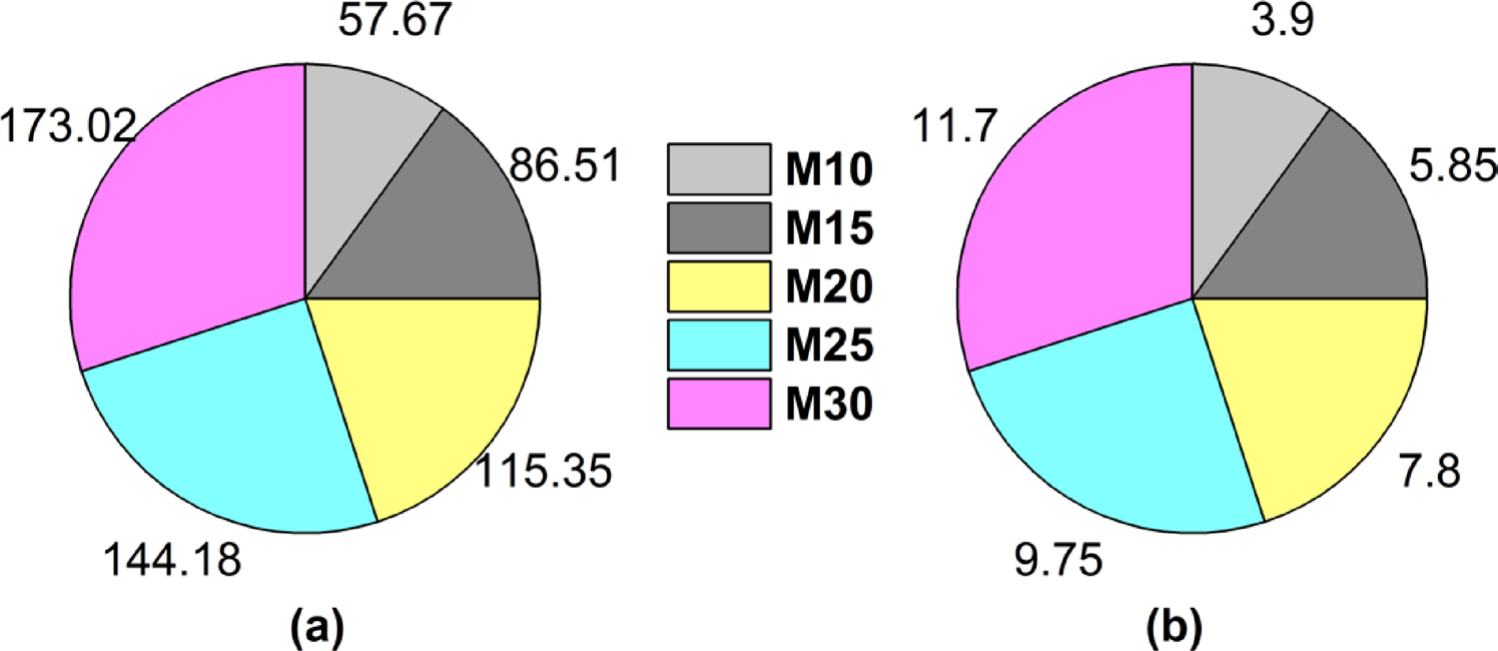
(**a**) Economic gain and (**b**) CO₂ Emission reduction with regards to calcined clay versus pozzolan.

The use of calcined clay as a replacement for natural pozzolan is considered both an economically and environmentally advantageous alternative. Pozzolan is sourced from the Beni-Saf region, located 782 km from the Ain Kebira cement company, whereas the clay is located only 3 km away from the cement company. This proximity significantly reduces transportation distances, thereby notably decreasing CO_2_ emissions and associated costs. Considering a transportation cost of €0.05 per kilometer^74^ and CO₂ emissions of 0.7375 kg per kilometer generated by trucks^75^. The adoption of calcined clay has enabled a reduction in CO_2_ emissions related to transportation by 28 to 173 kg per ton, depending on the substitution ratio ranging from 5 to 30%. Furthermore, the comparative cost analysis conducted in our study indicates that replacing natural pozzolan with calcined clay at substitution rates of 10%, 15%, 20%, 25%, and 30% results in cost savings of 3.90, 5.85, 7.80, 9.75, and 11.70 (in local currency) per ton of cement, respectively. These savings primarily arise from reduced transportation costs due to the proximity of clay deposits to the Ain El Kebira cement plant when compared to the more distant sources of natural pozzolan. Furthermore, calcined clay production is compatible with existing calcination infrastructure, which minimizes additional processing costs. Figure 14 clearly illustrates the environmental and economic benefits of using calcined clay when compared to natural pozzolan.

### Statistical analysis

To predict the compressive strength at 7 and 28 days, a comprehensive statistical analysis was conducted using a factorial design approach. This method allows for the evaluation of the influence of multiple factors and their interactions on the desired response. The analysis was carried out using JMP 14 PRO software, which provides robust tools for experimental design and data modeling. A full factorial design was employed, ensuring that all possible combinations of the factors under investigation were systematically tested.

**Fig. 15:**
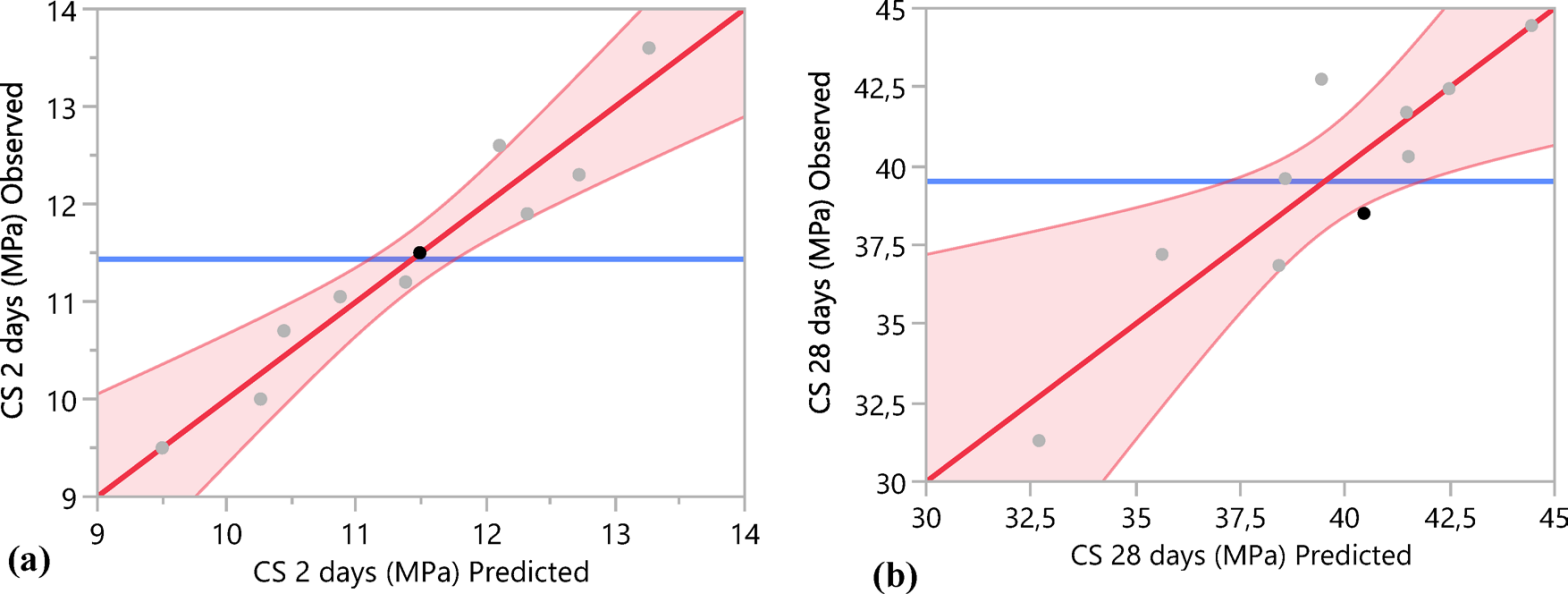
Correlation between the actual and predicted values; (**a**) compressive strength at 2 days and (**b**) compressive strength at 28 days.

The correlations between the actual and predicted values for compressive strength at 2 and 28 days are depicted in Fig. 15. The data points align closely with the fitting line in both instances. Furthermore, Table 9 provides a summary of the fit, revealing very high coefficients of determination (R^2^ = 0.81 and 0.93), which are indicative of a substantial agreement between the predicted and observed values. This high level of correlation implies that the predictive model is highly effective in capturing the key factors that influence compressive strength at these time intervals.

**Table 9:** Summary of fit.

	CS at 2 days (MPa)	CS at 28 days (MPa)
R^2^	0.93	0.81
Adjusted R^2^	0.9	0.72
RMSE	0.39	2
Mean of response	11.44	39.51
Observations	10	10

The analysis of variance (ANOVA) for each of the compressive strength responses is presented in Table 10. The significance of the models established in our study can be assessed using the distribution of the Fisher’s test. Indeed, for a 90% confidence interval, the Fisher ratios are equal to 27.75 and 8.71 for compressive strength at both 2 and 28 days respectively. These ratios are very high. Moreover, in this context, the probability values (Prob. > F) for all the models were less than 5%. The *p*-value confirms that there is at least one significant effect of a factor in each model.

**Table 10:** Analysis of variance (ANOVA).

	Source	Degree of freedom	Sum of squares	Mean square	F-ratio
CS (2 days)	Model	3	12.65	4.22	27.75
	Error	6	0.91	0.151	Prob. > F
	Total	9	13.56		0.0006*
CS (28 days)	Model	3	105.57	35.191	8.71
	Error	6	24.23	4.039	Prob. > F
	Total	9	129.81		0.0132*

The developed models are based on a full factorial design approach. The mathematical formulations are expected to be applicable to a wide range of mixtures with particle sizes ranging from 45 μm to 80 μm and calcined clay contents between 10 and 30% by mass of cement. The proposed mathematical models for predicting the compressive strength (cs) at both 2 and 28 days are as follows:

5 $$\begin{array}{c}\hbox{CS}\left(2\,\text{days}\right)=11.435-0.055\left(\frac{\text{Dmax}-62.5}{17.5}\right)-1.55\left(\frac{\text{C}-20}{10}\right)-0.325\left(\frac{\text{Dmax}-62.5}{17.5}\right)\left(\frac{\text{C}-20}{10}\right)\end{array}$$

6 $$\begin{array}{c}\hbox{CS}\left(28\,\text{days}\right)=39.51-0.94\left(\frac{\text{Dmax}-62.5}{17.5}\right)-3.95\left(\frac{\text{C}-20}{10}\right)-1.925\left(\frac{\text{Dmax}-62.5}{17.5}\right)\left(\frac{\text{C}-20}{10}\right)\end{array}$$

where Dmax and C represent the maximum particle size and the replacement level, respectively. This model is valid for Dmax ranging from 45 μm to 8 μm and a substitution rate between 10 and 30%. Table 11 presents the relative error between the experimental and the predicted values of compressive strength at 2 and 28 days. The obtained results demonstrate a strong correlation between the predicted and experimental values. The relative errors for the various formulations remain below 10%, highlighting the reliability and accuracy of the predictive model^76^.

**Table 11:** Comparison of the experimental and predicted values with the relative errors of compressive strength.

	Compressive strength at 2 days			Compressive strength at 28 days		
Mixtures	Experimental (MPa)	Predicted	Relative error (%)	Experimental (MPa)	Predicted	Relative error (%)
M10-80	13.26	13.6	2.54	44.45	44.45	0.01
M15-80	12.32	11.9	3.51	41.51	40.3	3
M20-80	11.38	11.2	1.61	38.57	39.6	2.6
M25-80	10.44	10.7	2.41	35.63	37.2	4.21
M30-80	9.51	9.5	0.05	32.7	31.3	4.46
M10-45	12.72	12.3	3.37	42.48	42.45	0.06
M15-45	12.1	12.6	3.95	41.46	41.7	0.57
M20-45	11.49	11.5	0.09	40.45	38.5	5.06
M25-45	10.88	11.05	1.56	39.44	42.75	7.75
M30-45	10.27	10	2.65	38.43	36.85	4.27

**Fig. 16:**
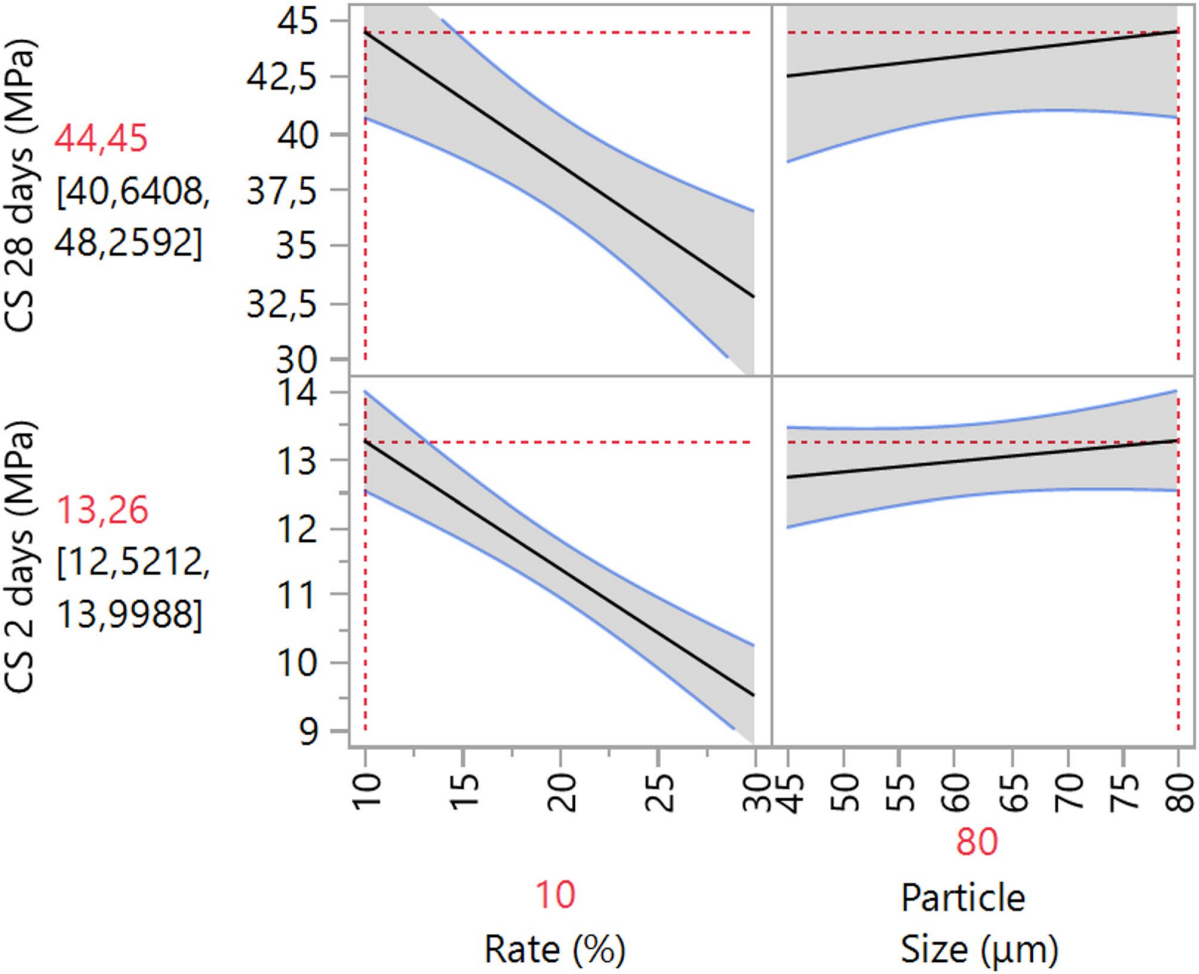
Main effect plot of the substitution ratio and particle size on compressive strength at both 2 and 28 days.

The influence of each parameter, specifically the fineness of grinding and the substitution ratio, is depicted in Fig. 16. The results indicate that increasing the substitution ratio of cement with calcined clay negatively impacts compressive strength, primarily due to a reduction in the formation of calcium silicate hydrate (C–S–H)^77^, which is critical for mechanical strength. Conversely, increasing the particle size of the calcined clay (i.e. decreasing the fineness) exerts a positive effect on mechanical strength. This enhancement in strength is attributed to improved particle distribution and reduced porosity, leading to a denser and more homogeneous microstructure. Therefore, while the higher substitution ratio diminishes C–S–H formation, the increased particle size contributes to a denser packing and better cohesion within the cementitious matrix, thereby improving the overall mechanical strength^63^.

**Fig. 17:**
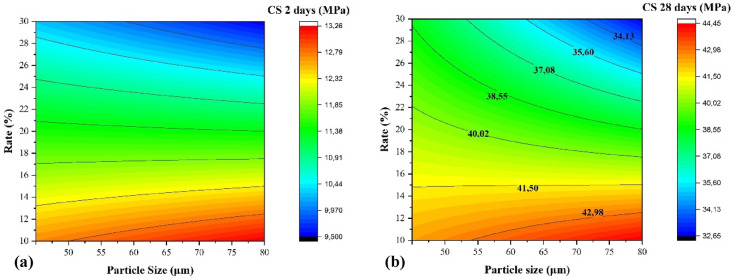
Linear Iso-response curve; (**a**) compressive strength at 2 days, (**b**) compressive strength at 28 days.

Figure 17 presents the iso-response curves for compressive strength at 2 and 28 days as a function of the maximum particle diameter of the grind and the substitution ratio of cement with calcined clay. Both curves demonstrate a similar trend: increasing the substitution ratio from 10 to 30% and decreasing the particle size from 80 μm to 45 μm result in a decline in mechanical strength. The higher substitution ratio decreases the clinker content, which is essential for the early formation of calcium silicate hydrate (C–S–H), the primary binder contributing to strength development. Furthermore, the finer particle size of calcined clay enhances its reactivity, but also increases the water demand, potentially leading to a less optimal water-to-cement ratio, which in turn adversely affects the hydration process^78^.

## Conclusion

In this study, an artificial pozzolan was thermally elaborated from local clay. Structural changes in the calcined clay due to heat treatment were characterized using FTIR analysis. The calcination temperature was determined based on FTIR and TGA results. The calcined clay was then ground to 45 µm and 80 µm and used as a replacement for cement in normal mortars. The pozzolanic activity of the pozzolan was evaluated by measuring its compressive strength. The effects of the fineness of the calcined clay and the ratio of cement replacement with calcined clay on the compressive and flexural strength of the mortars were examined. Based on the results of this experimental study, the following conclusions can be drawn:

- The optimal calcination temperature, at which nearly complete dihydroxylation of the material was achieved, is 700 °C. When kaolinitic clays are calcined at this temperature, they form an amorphous reactive phase, making kaolinitic calcined clays suitable for use as pozzolans,
- All mortars containing calcined clay as a replacement for cement achieve at least 75% of the compressive strength of the control mortar. According to ASTM C618, this calcined clay at 700 °C qualifies as an artificial pozzolan,
- At low replacement levels (10–15%), 80 µm fineness provides slightly higher early-age compressive strengths, such as 13.6 MPa at 2 days when compared to 12.3 MPa for 45 µm. However, at higher replacement levels (20–30%), 45 µm fineness demonstrates superior long-term performance, achieving 36.85 MPa at 28 days versus 31.3 MPa for 80 µm. This is attributed to the enhanced pozzolanic reactivity and densification of the matrix with finer particles, particularly at elevated replacement rates.
- The incorporation of calcined clay as a supplementary cementitious material demonstrates a consistent enhancement in flexural strength, with improvements ranging from 7 to 20% when compared to the reference mortar for substitution rates of up to 20%.
- The environmental analysis shows a consistent reduction in energy, CO_2_ emissions, and costs with increasing substitution ratios. The M30-45 and M30-80 series exhibit the highest reductions, with decreases of around 10.6% in energy, CO_2_, and costs. These results highlight the significant improvements in environmental and economic efficiency. Optimizing cement compositions offers clear benefits for sustainability and cost-effectiveness.
- The M15-45 and M15-80 mixtures (15% cement replacement with calcined clay of fineness below 45 µm and 80 µm, respectively), as well as the M20-45 and M20-80 mixtures, enable the production of CEM II/A-M 32.5 R. Meanwhile, the M30-45 and M25-80 mixtures are suitable for the production of CEM II/B-M 32.5 R, demonstrating the feasibility of expanding the range of cements that can be produced.
- The statistical analysis of compressive strength results at both 2 and 28 days yields determination coefficients (R^2^) of 0.93 and 0.81, respectively, indicating strong predictive accuracy. Additionally, the ANOVA results validate these findings, with *p*-values below 0.05, which confirms the statistical significance of the model.

Finally, the use of calcined clay as a partial replacement for cement in cementitious materials significantly reduces CO₂ emissions, thereby contributing to environmental protection. To further enhance the findings of this study, it would be beneficial to conduct additional investigations that address the following points: 1) a comparison between calcined and non-calcined clay to clearly assess the influence of calcination on mechanical performance; 2) microstructural analyses, such as X-ray diffraction (XRD), thermogravimetric analysis (TGA), differential thermal analysis (DTA), and scanning electron microscopy (SEM), to better understand the chemical phenomena and to correlate these results with the mechanical performance data; 3) a comprehensive life cycle analysis (LCA) of cement incorporating calcined clay, along with other supplementary materials such as pozzolan and glass powder, followed by a cost analysis to evaluate economic feasibility; and (4) a comparative evaluation of predictive modelling approaches, including full factorial design, artificial neural networks (ANN), and machine learning algorithms, to determine the most reliable method for forecasting mechanical performance based on key material and process parameters.

## Data Availability

Additional information and requests for materials should be addressed to J.S.
